# Association of Tumor-Associated Collagen Signature With Prognosis and Adjuvant Chemotherapy Benefits in Patients With Gastric Cancer

**DOI:** 10.1001/jamanetworkopen.2021.36388

**Published:** 2021-11-30

**Authors:** Dexin Chen, Hao Chen, Liangjie Chi, Meiting Fu, Guangxing Wang, Zhida Wu, Shuoyu Xu, Caihong Sun, Xueqin Xu, Liyan Lin, Jiaxin Cheng, Wei Jiang, Xiaoyu Dong, Jianping Lu, Jixiang Zheng, Gang Chen, Guoxin Li, Shuangmu Zhuo, Jun Yan

**Affiliations:** 1 Department of General Surgery, Guangdong Provincial Key Laboratory of Precision Medicine for Gastrointestinal Tumor, Nanfang Hospital, The First School of Clinical Medicine, Southern Medical University, Guangzhou, People’s Republic of China; 2 School of Science, Jimei University, Xiamen, People’s Republic of China; 3 Department of Gastrointestinal Surgery, Fujian Provincial Hospital, Teaching Hospital of Fujian Medical University, Fuzhou, People’s Republic of China; 4 Department of Gastroenterology, Guangdong Provincial Key Laboratory of Gastroenterology, Nanfang Hospital, The First School of Clinical Medicine, Southern Medical University, Guangzhou, People’s Republic of China; 5 Key Laboratory of OptoElectronic Science and Technology for Medicine of Ministry of Education, Fujian Normal University, Fuzhou, People’s Republic of China; 6 Department of Pathology, Fujian Medical University Cancer Hospital and Fujian Cancer Hospital, Fuzhou, People’s Republic of China

## Abstract

**Importance:**

The current TNM staging system provides limited information for prognosis prediction and adjuvant chemotherapy benefits for patients with gastric cancer (GC).

**Objective:**

To develop a tumor-associated collagen signature of GC (TACS_GC_) in the tumor microenvironment to predict prognosis and adjuvant chemotherapy benefits in patients with GC.

**Design, Setting, and Participants:**

This retrospective cohort study included a training cohort of 294 consecutive patients treated between January 1, 2012, and December 31, 2013, from Nanfang Hospital, Southern Medical University, People's Republic of China, and a validation cohort of 225 consecutive patients treated between October 1, 2010, and December 31, 2012, from Fujian Provincial Cancer Hospital, Fujian Medical University, People's Republic of China. In total, 146 collagen features in the tumor microenvironment were extracted with multiphoton imaging. A TACS_GC_ was then constructed using the least absolute shrinkage and selection operator Cox proportional hazards regression model in the training cohort. Data analysis was conducted from October 1, 2020, to April 30, 2021.

**Main Outcomes and Measures:**

The association of TACS_GC_ with disease-free survival (DFS) and overall survival (OS) was assessed. An independent external cohort was included to validate the results. Interactions between TACS_GC_ and adjuvant chemotherapy were calculated.

**Results:**

This study included 519 patients (median age, 57 years [IQR, 49-65 years]; 360 [69.4%] male). A 9 feature–based TACS_GC_ was built. A higher TACS_GC_ level was significantly associated with worse DFS and OS in both the training (DFS: hazard ratio [HR], 3.57 [95% CI, 2.45-5.20]; OS: HR, 3.54 [95% CI, 2.41-5.20]) and validation (DFS: HR, 3.10 [95% CI, 2.26-4.27]; OS: HR, 3.24 [95% CI, 2.33-4.50]) cohorts (continuous variable, *P* < .001 for all comparisons). Multivariable analyses found that carbohydrate antigen 19-9, depth of invasion, lymph node metastasis, distant metastasis, and TACS_GC_ were independent prognostic predictors of GC, and 2 integrated nomograms that included the 5 predictors were established for predicting DFS and OS. Compared with clinicopathological models that included only the 4 clinicopathological predictors, the integrated nomograms yielded an improved discrimination for prognosis prediction in a C index comparison (training cohort: DFS, 0.80 [95% CI, 0.73-0.88] vs 0.78 [95% CI, 0.71-0.85], *P* = .03; OS, 0.81 [95% CI, 0.75-0.88] vs 0.80 [95% CI, 0.73-0.86], *P* = .03; validation cohort: DFS, 0.78 [95% CI, 0.70-0.87] vs 0.76 [95% CI, 0.67-0.84], *P* = .006; OS, 0.78 [95% CI, 0.69-0.86] vs 0.75 [95% CI, 0.67-0.84], *P* = .002). Patients with stage II and III GC and low TACS_GC_ levels rather than high TACS_GC_ levels had a favorable response to adjuvant chemotherapy (DFS: HR, 0.65 [95% CI, 0.43-0.96]; *P* = .03; OS: HR, 0.55 [95% CI, 0.36-0.82]; *P* = .004; dichotomized variable, *P* < .001 for interaction for both comparisons).

**Conclusions and Relevance:**

The findings suggest that TACS_GC_ provides additional prognostic information for patients with GC and may distinguish patients with stage II and III disease who are more likely to derive benefits from adjuvant chemotherapy.

## Key Points

**Question:**

Can the accuracy of prognosis prediction be improved using a tumor-associated collagen signature of gastric cancer (TACS_GC_) to detect patients who are likely to benefit from adjuvant chemotherapy for GC?

**Findings:**

In this cohort study of 519 patients, a low TACS_GC_ level was associated with a better prognosis, and patients with stage II and III GC with a low TACS_GC_ level were more likely to benefit from adjuvant chemotherapy.

**Meaning:**

The findings suggest that TACS_GC_ may improve the accuracy of prognosis prediction and provide a helpful reference for decision-making regarding adjuvant chemotherapy in GC.

## Teaser

This cohort study of patients with gastric cancer examines the ability of a tumor-associated collagen signature to predict patient survival and adjuvant chemotherapy benefits.

## Introduction

Despite remarkable improvements in the diagnosis and treatment of gastric cancer (GC), it remains a major global health burden.^1^ The pathological staging system is the gold standard for treatment planning and prognosis prediction for patients with GC.^2^ Patients with advanced GC are advised to receive 5-fluorouracil–based adjuvant chemotherapy after radical surgery.^3,4,5^ However, significant variations in survival outcomes have been observed even among patients with the same pathological stage who receive similar treatment regimens.^6^ These findings indicate that the pathological staging system provides inadequate prognostic information and fails to accurately identify which patients might benefit from adjuvant chemotherapy. Thus, biomarkers that could improve the prognosis prediction and determine adjuvant chemotherapy benefits are urgently needed.

The extracellular matrix is important for regulating neoplastic progression and response to chemotherapy.^7^ As the main component of the extracellular matrix, collagen accounts for most of its functions.^8,9,10,11^ Increased collagen density enhances the invasiveness of tumor cells.^12,13^ Oriented collagen around tumor cells is also an indicator of disease progression.^14,15^ Moreover, stiff and dense collagen compresses intratumoral blood vessels, which induces hypoxia and impedes anticancer drug delivery.^8^ Thus, collagen alterations in the tumor microenvironment might provide information on prognosis and response to chemotherapy.

Multiphoton imaging has been widely applied in biological research.^16^ Multiphoton imaging, which combines 2-photon excitation fluorescence and second harmonic generation, is a stain-free method that shows comparable results to hematoxylin and eosin staining.^17^ Because of its physical origins, multiphoton imaging is sensitive for visualizing the collagen microstructure.^18^ High-dimensional quantitative collagen features, including morphologic and textural features, are therefore acquired from multiphoton images to describe collagen alterations.^19^

Integration of multiple features into a single signature shows a better performance than that of a single feature.^20,21^ Cox proportional hazards regression with the least absolute shrinkage and selection operator (LASSO) is a state-of-the-art method that is used for the regression of high-dimensional data for survival analysis.^22,23,24^ We used Cox proportional hazards regression with LASSO to construct a multiple collagen feature–based signature (ie, the tumor-associated collagen signature of GC [TACS_GC_]) to predict disease-free survival (DFS) and overall survival (OS) among patients with GC. Moreover, we investigated whether TACS_GC_ could distinguish patients who might benefit from adjuvant chemotherapy.

## Methods

### Study Design and Patients

The study design is shown in eFigure 1 in the Supplement. In this retrospective cohort study, we included 519 patients with resected GC from 2 medical centers. For training purposes, 294 consecutive patients were included from Nanfang Hospital, Southern Medical University, People's Republic of China, between January 1, 2012, and December 31, 2013. For validation purposes, 225 consecutive patients were included from Fujian Provincial Cancer Hospital, Fujian Medical University, People's Republic of China, between October 1, 2010, and December 31, 2012. The inclusion criteria were as follows: histologically diagnosed GC, radical gastrectomy with at least 15 lymph nodes harvested, availability of clinicopathological data, and complete postoperative follow-up. Patients treated with neoadjuvant chemotherapy, radiotherapy, or chemoradiotherapy were excluded. Data analysis was conducted from October 1, 2020, to April 30, 2021. This study was approved by the institutional review boards of Nanfang Hospital of Southern Medical University and Fujian Provincial Cancer Hospital of Fujian Medical University. Written informed consent was obtained from all patients before surgery, which contained a statement on the formalin-fixed, paraffin-embedded samples and clinicopathological data for scientific research. All data were deidentified. All procedures that involved human participants were performed in accordance with the Declaration of Helsinki.^25^ This study followed the Strengthening the Reporting of Observational Studies in Epidemiology (STROBE) reporting guideline and the Transparent Reporting of a Multivariable Prediction Model for Individual Prognosis or Diagnosis (TRIPOD) reporting guideline.

The baseline information, including age, sex, carcinoembryonic antigen level, cancer antigen 19-9 (CA 19-9) level, tumor location, tumor size, tumor differentiation, Lauren type, depth of invasion (T stage), lymph node metastasis (N stage), distant metastasis (M stage), TNM stage, and postoperative chemotherapy, was obtained from the archived data. The TNM stage was reclassified according to the eighth edition of the *AJCC Cancer Staging Manual* of the American Joint Committee on Cancer.^26^ Routine 5-fluorouracil–based adjuvant chemotherapy was initiated after surgery if patient conditions were suitable according to the National Comprehensive Cancer Network guidelines.

The primary outcomes were 5-year DFS and OS. The patients were followed up once every 3 months for the first 2 years after surgery, every 6 months for the next 3 years, and annually thereafter. The follow-up duration was measured from the time of surgery to the last follow-up, and survival status at the last follow-up was documented. Disease-free survival was defined as the interval between surgery and recurrence at any site or all-cause death, whichever came first. Overall survival was defined as the interval between surgery and death from any cause.

### Region of Interest Selection

The formalin-fixed, paraffin-embedded samples were prepared as 5-μm–thick sections for hematoxylin and eosin staining to determine the regions of interest, and the same regions on the other serial section were selected for multiphoton imaging. Two pathologists (Z.W. and L.L.) who were blinded to the clinical information independently reassessed the tumor region using a microscope. When the 2 pathologists had different opinions, a third pathologist (J.L.) was consulted and a consensus was reached through discussion. Finally, 5 regions of interest with a field of view of 500 × 500 μm per sample within the tumor region were randomly selected.

### Multiphoton Image Acquisition and Collagen Feature Extraction

The images were acquired from a commercial laser scanning multiphoton microscope (LSM 880, Zeiss) as previously reported (eMethods in the Supplement).^27^ The extraction of collagen features was performed via MATLAB 2015b (MathWorks).^9^ Four types of collagen features were extracted, including morphologic features and 3 types of textural features (histogram-based features, gray-level concurrence matrix–based features, and Gabor wavelet transform features). Detailed procedures are provided in the eMethods in the Supplement. Finally, a total of 146 features, including 12 morphologic features, 6 histogram-based features, 80 gray-level concurrence matrix–based features, and 48 Gabor wavelet transform features, were extracted (eTable 1 in the Supplement).

### Feature Selection and TACS_GC_ Construction

Cox proportional hazards regression with LASSO is an effective method to process high-dimensional predictors for survival analysis.^22,23^ This method uses an L1 penalty to shrink the coefficients to 0. The penalty parameter λ controls the strength of the penalty. If we reduce λ and relax the penalty, more predictors can enter the model. In this study, Cox proportional hazards regression with LASSO with 10-fold cross-validation was used to select the most predictive features in the training cohort,^22,23^ and a multiple feature–based TACS_GC_ was then constructed via a linear weighted combination of the selected features. The TACS_GC_ in the validation cohort was calculated by the selected features with their respective coefficients obtained from the training cohort.

### Association of the TACS_GC_ With Prognosis

An optimal cutoff value was identified via the maximally selected rank statistics to classify patients into high and low TACS_GC_ groups in the training cohort, and the same cutoff value was applied in the validation cohort.^28^ Survival differences between the high and low TACS_GC_ groups were compared, and the associations of TACS_GC_ with DFS and OS were evaluated.

### Development and Validation of the Integrated Nomograms for Prognosis Prediction

In the training cohort, the clinicopathological characteristics and TACS_GC_ were included in the univariate Cox proportional hazards regression analyses for DFS and OS. Variables with *P* < .05 were selected for the multivariable analyses. A backward stepwise regression was used to detect the independent predictors. Two integrated nomograms for the prediction of DFS and OS were developed based on the independent predictors.

To quantify the discrimination of the integrated nomograms, the C indexes were calculated, 5-year time-dependent receiver operator characteristic (ROC) curves were plotted, and the area under the ROC (AUROC) curves were computed.^29^ The C index used the survival time as response, and the time-independent AUROC curve used the binary survival status at a given time point as response. The former quantified how well the prediction model could order the survival times, and the latter quantified how well the prediction model could order the survival status at a given time point.^29^ To compare the agreement between the nomogram-predicted survival probabilities and the actual probabilities, calibration curves were generated.^30^ To evaluate the clinical usefulness, decision curve analysis was used to assess the net benefits at different threshold probabilities.^31,32^ The integrated nomograms were then applied in the validation cohort to validate the discrimination, calibration, and clinical usefulness.^33^

### Incremental Value of the TACS_GC_ for Prognosis Prediction

To evaluate the incremental value of the TACS_GC_ to clinicopathological risk factors for prognosis prediction, 2 clinicopathological models, including independent clinicopathological risk factors for the prediction of DFS and OS, were built in the training cohort and then applied in the validation cohort. The incremental value of the TACS_GC_ to the clinicopathological models and TNM staging system was assessed with respect to discrimination, reclassification, and clinical usefulness.^33^

### Statistical Analysis

Continuous variables were compared by the independent sample, unpaired *t* test if normally distributed or the Mann-Whitney *U* test if nonnormally distributed. When a categorical variable was compared, if the table was a 2 × 2 contingency table with at least 1 expected cell count less than 5, the Fisher exact test was calculated or the χ^2^ test was used; if the table was a 2 × C (C > 2) contingency table, the χ^2^ test was performed. C indexes and time-independent AUROC curves were compared by the *z* score test and DeLong test, respectively. The Kaplan-Meier method and log-rank test were used to estimate DFS and OS, and Cox proportional hazards regression was conducted to compute the hazard ratios (HRs) with 95% CIs. Interactions between the TACS_GC_ level (high vs low) and adjuvant chemotherapy (yes vs no) were tested by Cox proportional hazards regression. All statistical analyses were performed using R software, version 3.6.2 (R Foundation for Statistical Computing) and SPSS software, version 19.0 (IBM Inc). A 2-sided *P* < .05 was considered statistically significant.

## Results

### Clinicopathological Characteristics

Of the 519 patients included in the study (median age, 57 years [IQR, 49-65 years]; 360 [69.4%] male), 294 consecutive patients (median age, 57 years [IQR, 49-64 years]; 208 [70.7%] male) were included in the training cohort and 225 consecutive patients (median age, 58 years [IQR, 51-65 years]; 152 [67.6%] male) were included in the validation cohort. The clinicopathological characteristics of all patients are summarized in Table 1. No significant difference in the clinicopathological characteristics was found between the 2 cohorts. The clinicopathological characteristics between patients with and without complete data were similar (eTable 2 in the Supplement).

**Table 1.** Clinicopathological Characteristics of Patients in the Training and Validation Cohorts^a^

Characteristic	Training cohort (n = 294)	Validation cohort (n = 225)	*P* value
Age, y			
≤60	185 (62.9)	134 (59.6)	.43
>60	109 (37.1)	91 (40.4)	
Age, median (IQR), y^b^	57 (49-64)	58 (51-65)	.55
Sex			
Female	86 (29.3)	73 (32.4)	.43
Male	208 (70.7)	152 (67.6)	
CEA level^c^			
Normal	245 (83.3)	197 (87.6)	.18
Elevated	49 (16.7)	28 (12.4)	
CA 19-9 level^d^			
Normal	250 (85.0)	185 (82.2)	.39
Elevated	44 (15.0)	40 (17.8)	
Tumor location			
Fundus of stomach	70 (23.8)	43 (19.1)	.39
Body of stomach	51 (17.3)	45 (20.0)	
Antrum of stomach	173 (58.9)	137 (60.9)	
Tumor size, cm			
≤4	170 (57.8)	113 (50.2)	.09
>4	124 (42.2)	112 (49.8)	
Tumor differentiation			
Well	20 (6.8)	14 (6.2)	.82
Moderate	63 (21.4)	44 (19.6)	
Poor and undifferentiated	211 (71.8)	167 (74.2)	
Lauren type			
Intestinal type	137 (46.6)	98 (43.6)	.49
Diffused or mixed type	157 (53.4)	127 (56.4)	
Depth of invasion			
T1	61 (20.7)	30 (13.3)	.11
T2	26 (8.8)	20 (8.9)	
T3	30 (10.2)	18 (8.0)	
T4a	155 (52.7)	131 (58.2)	
T4b	22 (7.6)	26 (11.6)	
Lymph node metastasis			
N0	135 (45.9)	86 (38.2)	.46
N1	52 (17.7)	42 (18.7)	
N2	44 (15.0)	41 (18.2)	
N3a	39 (13.3)	32 (14.2)	
N3b	24 (8.1)	24 (10.7)	
Distant metastasis			
M0	283 (96.3)	216 (96.0)	.88
M1	11 (3.7)	9 (4.0)	
TNM stage			
I	69 (23.5)	32 (14.2)	.07
II	82 (27.9)	70 (31.1)	
III	132 (44.9)	114 (50.7)	
IV	11 (3.7)	9 (4.0)	
Adjuvant chemotherapy			
Yes	173 (58.8)	132 (58.7)	.97
No	121 (41.2)	93 (41.3)	

Abbreviations: CA, cancer antigen; CEA, carcinoembryonic antigen.

SI conversion factor: To convert CEA to micrograms per liter, multiply by 1.0.

^a^ Data are presented as number (percentage) of patients unless otherwise indicated.

^b^ The median difference in age between training and validation cohorts was 1 year (95% CI, −1 to 3 years).

^c^ For CEA, elevated indicates 5 ng/mL or greater; normal, less than 5 ng/mL.

^d^ For CA 19-9, elevated indicates 37 U/mL or greater; normal, less than 37 U/mL.

### Construction of the TACS_GC_

**Figure 1.**
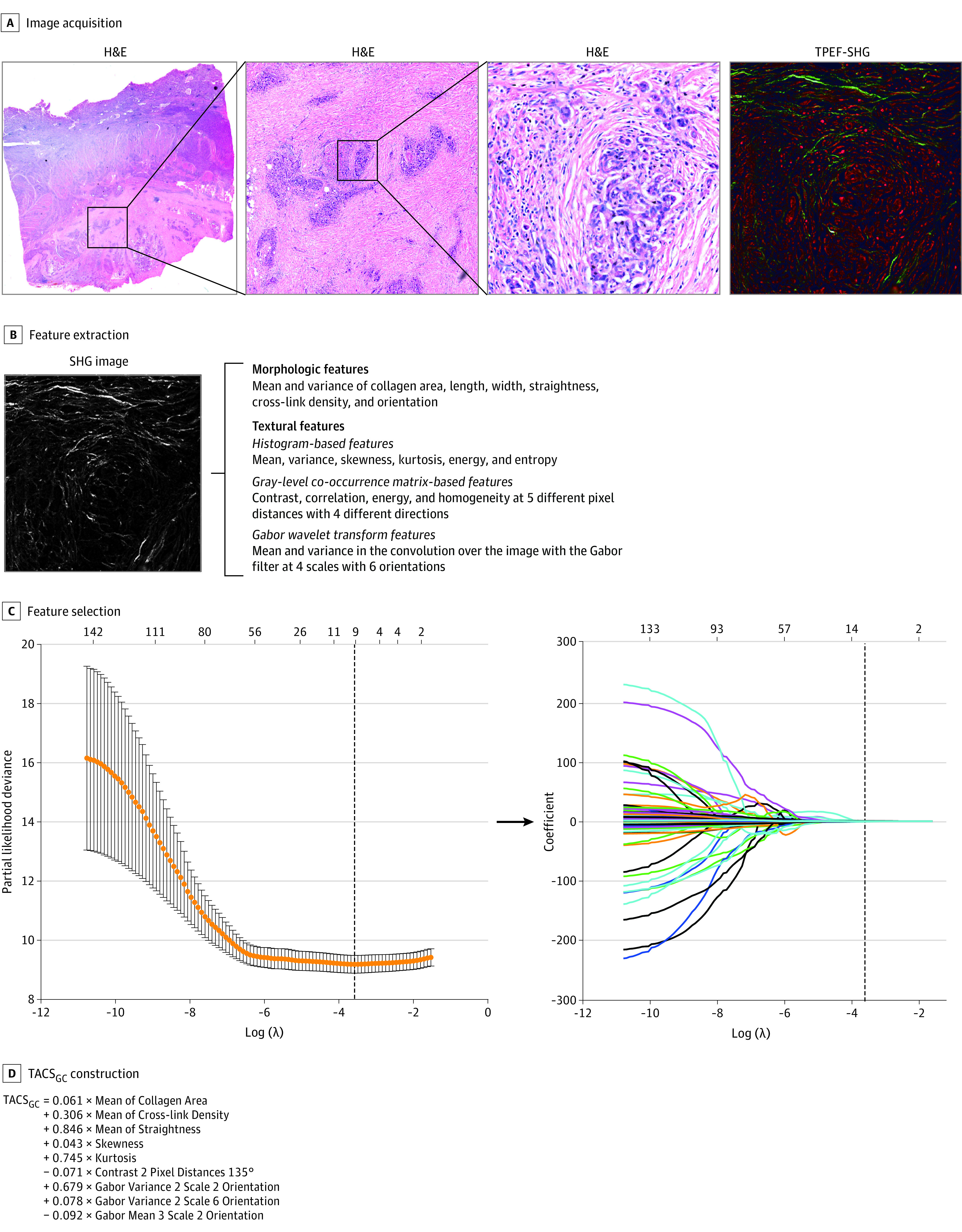
Schematic of Tumor-Associated Collagen Signature of Gastric Cancer (TACS_GC_) A, Hematoxylin and eosin (H&E) and 2-photon excitation fluorescence (TPEF) and second harmonic generation (SHG) combined images. A representative region of interest with a field of view of 500 × 500 μm was selected in the H&E staining image. The corresponding multiphoton imaging was obtained. B, The SHG image was chosen for collagen feature extraction, including morphologic and textural features. C, Collagen feature selection used the least absolute shrinkage and selection operator Cox proportional hazards regression model with 10-fold cross-validation. Dashed vertical lines represent the optimal log(λ) value after the last step. D, Construction of the TACS_GC_ was based on the selected collagen features.

The construction framework of the TACS_GC_ is presented in Figure 1. A TACS_GC_ that included 9 collagen features was constructed (eFigure 2 in the Supplement). On the basis of the training cohort, an optimal cutoff value of 2.81 was determined, and all included patients were divided into high and low TACS_GC_ groups (eFigure 3 in the Supplement). The distribution of TACS_GC_ with the corresponding survival status is shown in eFigure 4 in the Supplement. Patients with higher TACS_GC_ values were more likely to experience disease recurrence or death. No significant difference was found in the TACS_GC_ values between the training (median, 2.47 [IQR 2.08-2.91]) and validation (median, 2.55 [IQR 2.08-2.96]) cohorts (median difference, −0.05; 95% CI, −0.15 to 0.05; *P* = .23). The TACS_GC_ level was significantly associated with tumor differentiation, depth of invasion, lymph node metastasis, and distant metastasis (eTables 3-5 in the Supplement).

### Association of the TACS_GC_ With Prognosis

**Figure 2.**
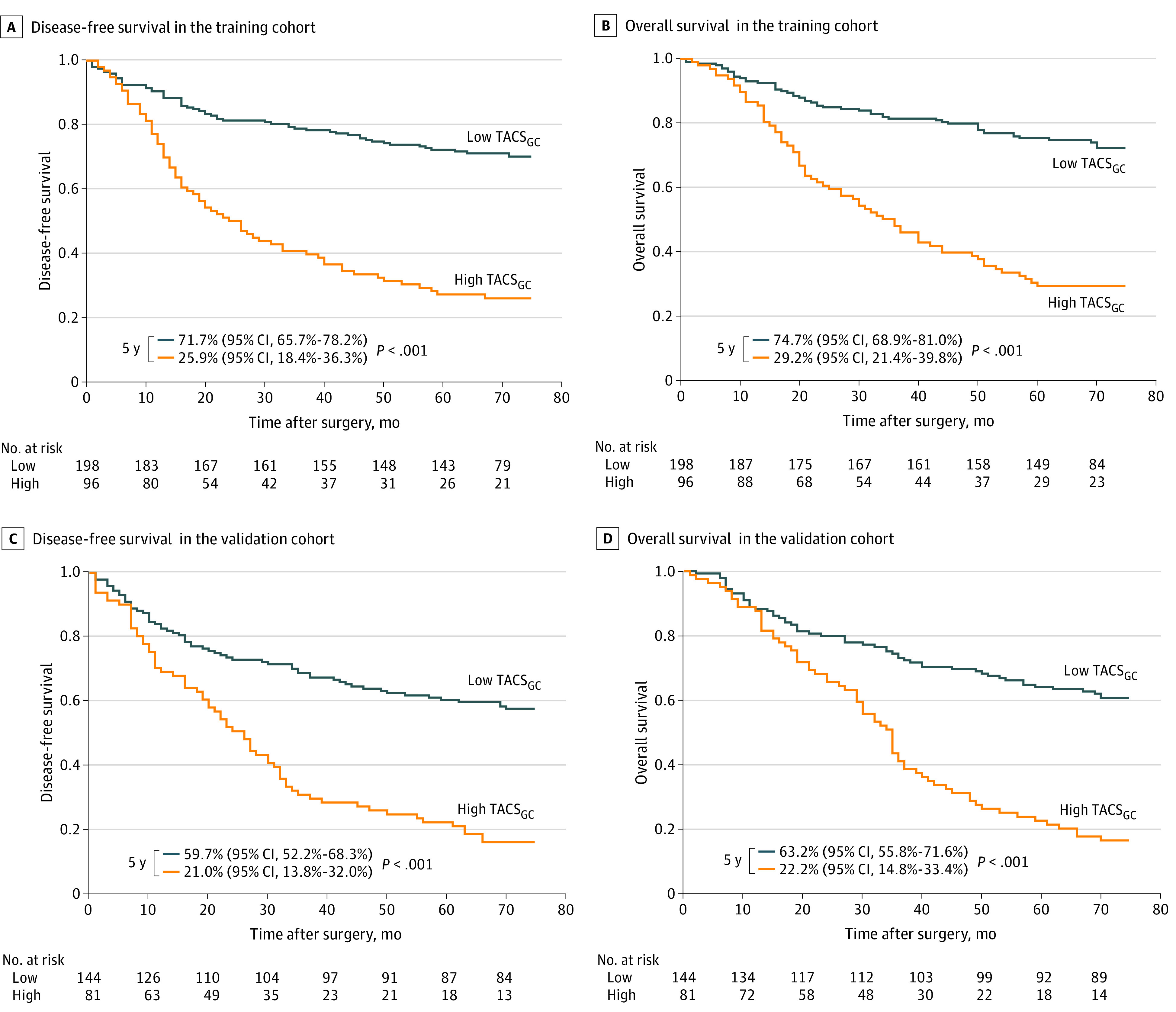
Kaplan-Meier Survival Analysis of the Training and Validation Cohorts Grouped by the Tumor-Associated Collagen Signature of Gastric Cancer (TACS_GC_)

In the training cohort, the 5-year DFS and OS were 71.7% (95% CI, 65.7%-78.2%) and 74.7% (95% CI, 68.9%-81.0%), respectively, in the low TACS_GC_ group. In the high TACS_GC_ group, the 5-year DFS and OS were significantly decreased to 25.9% (95% CI, 18.4%-36.3%) and 29.2% (95% CI, 21.4%-39.8%), respectively (Figure 2A and B). These results were further verified in the validation cohort (Figure 2C and D). The TACS_GC_ remained a significant prognostic indicator after stratification by clinicopathological variables, demonstrating the independent association of the TACS_GC_ with prognosis (eFigures 5 and 6 in the Supplement).

### Development and Validation of the Integrated Nomograms for Prognosis Prediction

Univariate Cox proportional hazards regression analyses showed that TACS_GC_ was significantly associated with worse DFS and OS in both the training (DFS: HR, 3.57 [95% CI, 2.45-5.20]; OS: HR, 3.54 [95% CI, 2.41-5.20]; *P* < .001 for both comparisons) and validation (DFS: HR, 3.10 [95% CI, 2.26-4.27]; OS: HR, 3.24 [95% CI, 2.33-4.50]; *P* < .001 for both comparisons) cohorts (eTables 6 and 7 in the Supplement). Backward stepwise multivariable Cox proportional hazards regression analyses found that the TACS_GC_ was still independently associated with DFS and OS after adjustment for CA 19-9, T stage, N stage, and M stage in the training (DFS: HR, 2.03 [95% CI, 1.40-2.95]; OS: HR, 1.95 [95% CI, 1.33-2.88]; *P* < .001 for both comparisons) and validation (DFS: HR, 2.61 [95% CI, 1.82-3.76]; OS: HR, 2.39 [95% CI, 1.69-3.38]; *P* < .001 for both comparisons) cohorts (Table 2).

**Table 2.** Multivariable Cox Proportional Hazards Regression Analyses for Disease-Free Survival and Overall Survival

Variable	Disease-free survival			Overall survival		
	HR (95% CI)	*P* value	Overall *P* value^a^	HR (95% CI)	*P* value	Overall *P* value^a^
**Training cohort**						
TACS_GC_^b^	2.03 (1.40-2.95)	<.001	NA	1.95 (1.33-2.88)	<.001	NA
CA 19-9 (elevated vs normal)	1.62 (1.04-2.50)	.03	NA	1.62 (1.03-2.53)	.04	NA
Depth of invasion						
T1	1 [Reference]	NA	.04	1 [Reference]	NA	.02
T2	1.50 (0.45-5.00)	.51		2.13 (0.56-8.12)	.27	
T3	2.73 (1.00-7.49)	.05		3.25 (1.00-10.58)	.05	
T4a	3.25 (1.34-7.86)	.009		4.48 (1.56-12.85)	.005	
T4b	3.99 (1.45-10.96)	.007		6.28 (1.96-20.10)	.002	
Lymph node metastasis						
N0	1 [Reference]	NA	<.001	1 [Reference]	NA	<.001
N1	1.43 (0.79-2.60)	.24		1.55 (0.83-2.90)	.17	
N2	2.79 (1.59-4.90)	<.001		3.00 (1.66-5.40)	<.001	
N3a	4.86 (2.79-8.49)	<.001		4.76 (2.66-8.48)	<.001	
N3b	7.02 (3.79-13.00)	<.001		7.22 (3.82-13.64)	<.001	
Distant metastasis (M1 vs M0)	2.03 (1.03-4.00)	.04	NA	2.12 (1.06-4.22)	.03	NA
**Validation cohort**						
TACS_GC_	2.61 (1.82-3.76)	<.001	NA	2.39 (1.69-3.38)	<.001	NA
CA 19-9 (elevated vs normal)	1.87 (1.22-2.87)	.004	NA	1.60 (1.03-2.47)	.04	NA
Depth of invasion						
T1	1 [Reference]	NA	.05	1 [Reference]	NA	.03
T2	1.82 (0.59-5.61)	.30		1.89 (0.61-5.80)	.27	
T3	2.09 (0.69-6.32)	.19		2.26 (0.75-6.81)	.15	
T4a	2.40 (0.94-6.13)	.07		2.77 (1.09-7.05)	.03	
T4b	4.06 (1.48-11.18)	.007		4.46 (1.63-12.19)	.004	
Lymph node metastasis						
N0	1 [Reference]	NA	<.001	1 [Reference]	NA	<.001
N1	2.27 (1.24-4.13)	.007		2.41 (1.33-4.35)	.004	
N2	2.76 (1.50-5.07)	.001		3.21 (1.76-5.87)	<.001	
N3a	3.60 (1.92-6.74)	<.001		4.44 (2.36-8.34)	<.001	
N3b	7.58 (4.04-14.20)	<.001		7.41 (3.97-13.84)	<.001	
Distant metastasis (M1 vs M0)	2.14 (0.96-4.73)	.06	NA	2.01 (0.90-4.52)	.09	NA

Abbreviations: CA, cancer antigen; HR, hazard ratio; NA, not applicable; TACS_GC_, tumor-associated collagen signature of gastric cancer.

^a^ Overall *P* values of the specified variables were determined using the Wald test.

^b^ TACS_GC_ is a continuous variable with a per-unit increase of 1.

On the basis of the multivariable Cox proportional hazards regression analyses in the training cohort, 2 integrated nomograms for the prediction of DFS and OS, including TACS_GC_, CA 19-9, T stage, N stage, and M stage, were developed (eFigure 7 in the Supplement). The integrated nomograms displayed C indexes of 0.80 (95% CI, 0.73-0.88) for DFS and 0.81 (95% CI, 0.75-0.88) for OS in the training cohort. In the validation cohort, the C indexes were 0.78 (95% CI, 0.70-0.87) for DFS and 0.78 (95% CI, 0.69-0.86) for OS. The calibration curves of the 2 integrated nomograms showed satisfactory agreement between the nomogram-predicted survival and actual survival in both the training and validation cohorts (eFigure 8 in the Supplement).

### Incremental Value of the TACS_GC_ for Prognosis Prediction

To evaluate the incremental value of the TACS_GC_ for prognosis prediction, 2 clinicopathological models, including T stage, N stage, M stage, and CA 19-9, for the prediction of DFS and OS were built (eTable 8 in the Supplement). Compared with the integrated nomograms, the clinicopathological models showed significantly decreased C indexes of 0.78 (95% CI, 0.71-0.85; *P* = .03) for DFS and 0.80 (95% CI, 0.73-0.86; *P* = .03) for OS in the training cohort. Similarly, significantly reduced C indexes of 0.76 (95% CI, 0.67-0.84; *P* = .006) for DFS and 0.75 (95% CI, 0.67-0.84; *P* = .002) for OS were detected in the validation cohort. In addition, the C indexes of the TNM staging system alone for DFS and OS were also reduced (eTable 9 in the Supplement). Moreover, significantly improved 5-year AUROCs were also found in the integrated nomograms for DFS and OS compared with the clinicopathological models (eFigure 9 in the Supplement). In addition, the integrated nomograms yielded net reclassification improvement values of 0.21 (95% CI, 0.05-0.33; *P* = .01) for DFS and 0.21 (95% CI, 0.02-0.32; *P* = .03) for OS to the clinicopathological models in the training cohort. Similar results were observed in the validation cohort (eTable 10 in the Supplement). Finally, the decision curve analysis showed that the integrated nomograms had a higher net benefit than the clinicopathological models and TNM staging system across most of the range of threshold probabilities (eFigure 10 in the Supplement).

### Association of the TACS_GC_ Level With Adjuvant Chemotherapy Benefits

**Figure 3.**
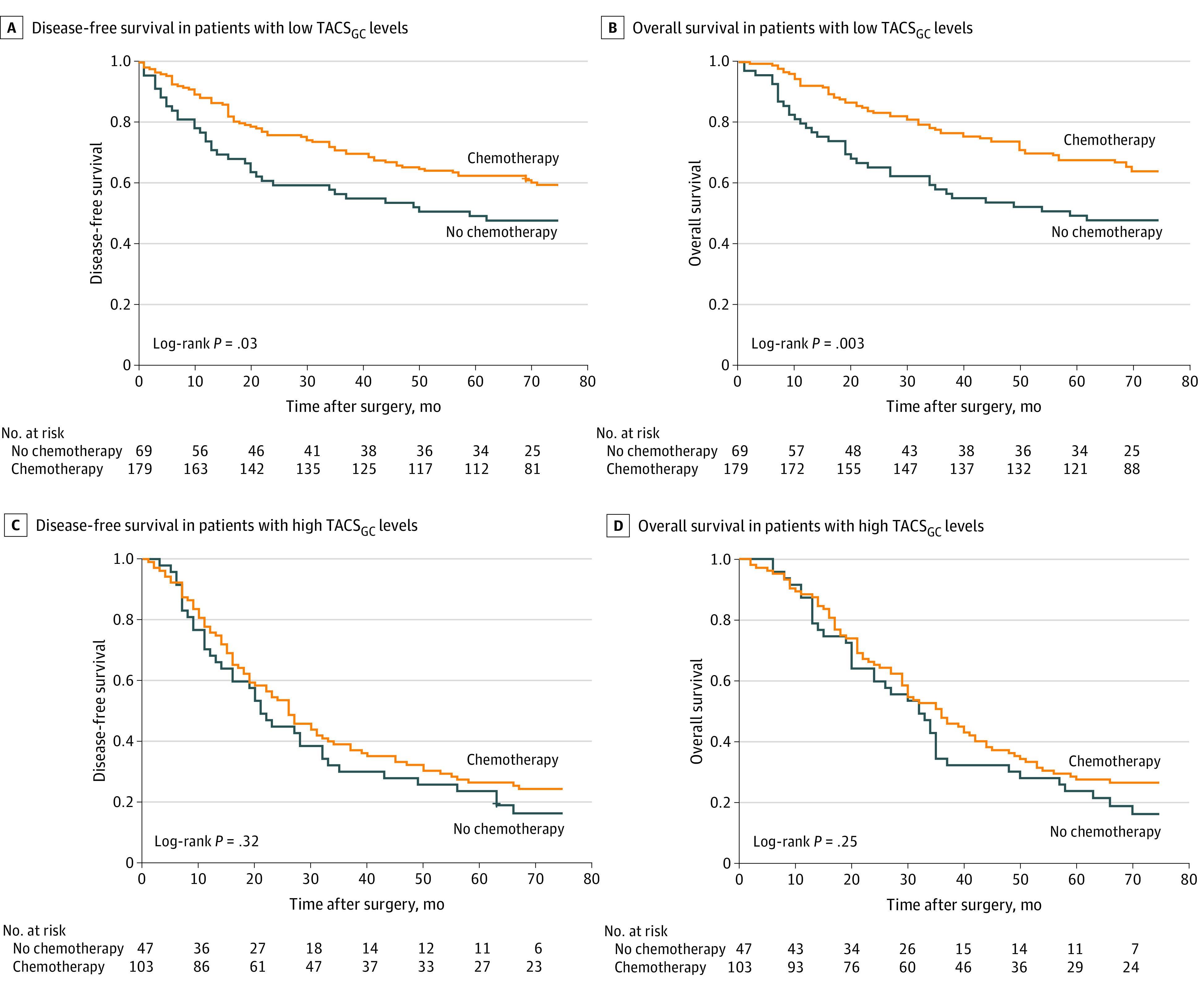
Association of Adjuvant Chemotherapy With Disease-Free Survival and Overall Survival in Patients With Stage II and III Gastric Cancer TACS_GC_ indicates tumor-associated collagen signature of gastric cancer.

The clinicopathological characteristics of patients with stage II and III GC according to adjuvant chemotherapy are listed in eTable 11 in the Supplement. An interaction test between the TACS_GC_ level and adjuvant chemotherapy indicated that patients with low TACS_GC_ levels had a superior benefit from adjuvant chemotherapy compared with patients with high TACS_GC_ levels (*P* < .05 for all comparisons) (eTable 12 in the Supplement). Patients with stage II and III GC and low TACS_GC_ levels rather than high TACS_GC_ levels had a favorable response to adjuvant chemotherapy (DFS: HR, 0.65 [95% CI, 0.43-0.96]; *P* = .03; OS: HR, 0.55 [95% CI, 0.36-0.82]; *P* = .004; dichotomized variable, *P* < .001 for interaction for both comparisons) (Figure 3). Subgroup analyses according to the TNM stage demonstrated that patients with low TACS_GC_ levels could experience significant survival benefits in the subgroups with stage II and III GC, but for patients with high TACS_GC_ levels, the survival benefits were less clear in the subgroup with stage II GC and were clinically small in the subgroup with stage III GC (eFigure 11 and 12 in the Supplement).

## Discussion

An accurate prediction of prognosis and survival benefits from adjuvant chemotherapy in patients with GC is integral to treatment decision-making. In this study, we constructed a TACS_GC_ in the tumor microenvironment of GC based on multiphoton imaging and demonstrated that the TACS_GC_ was significantly associated with prognosis of GC. Moreover, by incorporating TACS_GC_ into clinicopathological models, we found that the TACS_GC_ could provide additional prognostic information. Furthermore, we showed that the TACS_GC_ was a potential indicator of survival benefits associated with adjuvant chemotherapy in patients with stage II and III GC.

The construction of the TACS_GC_ was determined mainly by 3 key factors. First, the use of multiphoton imaging could specifically visualize the morphologic features of collagen because of its physical origins.^16^ Compared with the pathological staining methods of collagen, such as Masson trichrome staining, multiphoton imaging is acquired based on the intrinsic signals, thus avoiding additional reagents and batch-to-batch variations, which yields more sensitive information about collagen and ensures reproducible results.^34^ Second, an objectively quantitative method for the extraction of collagen features is essential for describing collagen alterations in the tumor microenvironment. For this purpose, we established a standardized quantification framework for the quantitative measurement of high-dimensional collagen features from multiphoton imaging.^11,35^ The third factor was a statistical model that could integrate high-dimensional features into a single signature. Cox proportional hazards regression with LASSO, a state-of-the-art statistical model that has satisfactory performance for dealing with high-dimensional data, was therefore used.^22,23,36^ After considering the 3 key factors, the TACS_GC_ was constructed.

Collagen is usually organized as an isotropic meshwork in normal extracellular matrix but is realigned during tumor invasion.^37^ Provenzano et al^13^ revealed that increased collagen density accelerated tumorigenesis, local invasion, and metastasis, causally linking increased stromal collagen to tumor formation and progression. A recent investigation^10^ also reported that a collagen-rich tumor microenvironment facilitated the early metastatic onset of tumor cells via interaction with integrin-α_2_, thus promoting cell migration and anoikis resistance. In this study, a higher TACS_GC_ level was an indicator of poorer prognosis. According to the calculation formula, TACS_GC_ was positively correlated with collagen area, collagen straightness, and collagen cross-link density, which was in accordance with previous findings.^7^ However, the underlying mechanisms remain unclear. Therefore, additional work should focus on the underlying mechanisms of the interaction of tumor cells and collagen.

In addition to the morphologic features, textural features have also been measured for a more comprehensive description of collagen organization.^38^ Currently, a common consensus about what types of textural features should be included to represent collagen alterations has not yet been reached. However, the textural features mainly included first-order statistics (eg, histogram-based features), second-order statistics (eg, gray-level concurrence matrix–based features), and wavelet transformation (eg, Gabor wavelet transform features),^38,39,40^ which have been proven to have powerful potential for disease diagnosis. Stromal collagen information could be robustly measured and quantified via the combination of morphologic and textural features.^41^ Although most published studies^38,39,40,41^ only assessed the correlation of textural features and patient outcomes, we assumed that there is an underlying molecular assignment for different textural features.

According to the so-called seed and soil hypothesis, cancer metastasis originated from the intricate interaction between tumor cells and their microenvironment.^42^ The tumor cells play the principal role and the tumor microenvironment plays the secondary role during the process of cancer metastasis.^37^ In the integrated nomograms, CA 19-9, depth of invasion, lymph node metastasis, and distant metastasis represented the tumor cells, and the TACS_GC_ represented the structural tumor microenvironment. Therefore, despite prognosis prediction reaching statistical significance, the incremental value of TACS_GC_ was small. From the perspective of clinicians, the TACS_GC_ provided additional prognostic information and helped researchers to understand the interactions between tumor cells and their structural microenvironment; therefore, the TACS_GC_ was clinically relevant and worth investigation.

Adjuvant chemotherapy is the standard treatment for patients with stage II and III GC to improve survival outcomes.^2,3,4,5,6^ However, many patients with stage II and III GC do not experience survival benefits from adjuvant chemotherapy, indicating that some patients might be overtreated because their tumors are not sensitive to the given type of chemotherapy.^43^ Xiong et al^44^ proposed that increased intratumoral collagen deposition was correlated with resistance to chemotherapy and with reduced survival in patients with breast cancer. In the present study, improved oncologic outcomes were detected in patients with low TACS_GC_ levels who received adjuvant chemotherapy but not in patients with high TACS_GC_ levels. Therefore, the TACS_GC_ level might be helpful for identifying patients with stage II and III GC who might benefit from adjuvant chemotherapy.

Multiphoton imaging has been recognized as a novel method for optical biopsy of samples.^17^ Because of the comparable results between multiphoton imaging and hematoxylin and eosin staining, pathologists or clinicians could differentiate the cancerous and normal tissues of the stomach after minimal training.^45,46^ Routine formalin fixation and paraffin embedding were reported to have negligible influence on multiphoton image acquisition,^47^ and approximately 10 minutes is needed to perform multiphoton imaging; thus, multiphoton imaging and TACS_GC_ are promising for clinical application. We believe that clinicians could obtain TACS_GC_ in the near future using multiphoton imaging, and the integrated nomograms could be used in clinical practice.

### Limitations

This study has limitations. First, it was retrospective in nature, and all specimens were acquired from 2 medical centers in China; thus, potential bias was inevitable. A prospective, multicenter trial is needed to validate the performance of TACS_GC_. Second, the underlying mechanism of the TACS_GC_ for the prediction of prognosis and adjuvant chemotherapy benefits remains unclear; therefore, further investigations are needed to better understand the role of TACS_GC_ in tumor progression and chemotherapy response.

## Conclusions

The findings suggest that TACS_GC_ could provide additional prognostic information to the TNM staging system for prognosis prediction of GC. Moreover, the TACS_GC_ value might be useful to distinguish patients with stage II and III GC who might benefit from adjuvant chemotherapy. Thus, the TACS_GC_ might be helpful for patient counseling, decision-making regarding adjuvant chemotherapy, and follow-up scheduling.

## Notes

Supplement. **eMethods.** Supplemental Methods **eFigure 1.** Study Design of This Study **eFigure 2.** Collagen Feature Selection Was Performed Using the LASSO-Cox Regression Model **eFigure 3.** The Optimal Cutoff Value for TACS_GC_ Was Determined Using Maximally Selected Rank Statistics **eFigure 4.** Distribution of the TACS_GC_ With Corresponding Survival Status in Training and Validation Cohorts **eFigure 5.** Kaplan-Meier Survival Analysis of DFS for All 519 Patients According to the TACS_GC_ Level Stratified by Clinicopathological Variables **eFigure 6.** Kaplan-Meier Survival Analysis of OS for All 519 Patients According to the TACS_GC_ Level Stratified by Clinicopathological Variables **eFigure 7.** Integrated Nomograms for DFS and OS **eFigure 8.** Calibration Curves of the Integrated Nomograms for DFS and OS **eFigure 9.** Time-Independent ROC Curves at 5 Years of Different Models for DFS and OS **eFigure 10.** Decision Curve Analysis of Each Model for Predicting Survival **eFigure 11.** Adjuvant Chemotherapy Benefits in Stage II GC Patients in Terms of DFS and OS **eFigure 12.** Adjuvant Chemotherapy Benefits in Stage III GC Patients in Terms of DFS and OS **eTable 1.** Summarization of the 146 Extracted Collagen Features **eTable 2.** Comparison of Clinicopathological Characteristics Between Patients With and Without Complete Data **eTable 3.** Clinicopathological Characteristics of Patients Grouped by TACS_GC_ in the Training Cohort **eTable 4.** Clinicopathological Characteristics of Patients Grouped by TACS_GC_ in the Validation Cohort **eTable 5.** Clinicopathological Characteristics of Patients Grouped by TACS_GC_ in the Total Cohort **eTable 6.** Univariate Cox Regression Analyses of DFS and OS in Training Cohort **eTable 7.** Univariate Cox Regression Analyses of DFS and OS in Validation Cohort **eTable 8.** Multivariable Cox Regression Analyses of DFS and OS Without TACS_GC_ **eTable 9.** C-index Comparison of Integrated Nomogram With Other Prediction Models **eTable 10.** The Net Reclassification Improvement of Adding the TACS_GC_ to the Clinicopathological Model **eTable 11.** Clinicopathological Characteristics of Stage II and III Patients According to Chemotherapy **eTable 12.** Adjuvant Chemotherapy Interaction With the TACS_GC_ for DFS and OS in Stage II and III Patients
eReferences

## References

[1] GBD 2017 Stomach Cancer Collaborators. The global, regional, and national burden of stomach cancer in 195 countries, 1990-2017: a systematic analysis for the Global Burden of Disease study 2017. Lancet Gastroenterol Hepatol. 2020;5(1):42-54. doi:10.1016/S2468-1253(19)30328-0 31648970 PMC7033564

[2] Ajani JA, D’Amico TA, Almhanna K, . Gastric cancer, version 3.2016, NCCN clinical practice guidelines in oncology. J Natl Compr Canc Netw. 2016;14(10):1286-1312. doi:10.6004/jnccn.2016.0137 27697982

[3] Bang YJ, Kim YW, Yang HK, ; CLASSIC Trial Investigators. Adjuvant Capecitabine and Oxaliplatin for Gastric Cancer After D2 Gastrectomy (CLASSIC): a phase 3 open-label, randomised controlled trial. Lancet. 2012;379(9813):315-321. doi:10.1016/S0140-6736(11)61873-4 22226517

[4] Sasako M, Sakuramoto S, Katai H, . Five-year outcomes of a randomized phase III trial comparing adjuvant chemotherapy with S-1 versus surgery alone in stage II or III gastric cancer. J Clin Oncol. 2011;29(33):4387-4393. doi:10.1200/JCO.2011.36.5908 22010012

[5] Noh SH, Park SR, Yang HK, ; CLASSIC Trial Investigators. Adjuvant Capecitabine Plus Oxaliplatin for Gastric Cancer After D2 Gastrectomy (CLASSIC): 5-year follow-up of an open-label, randomised phase 3 trial. Lancet Oncol. 2014;15(12):1389-1396. doi:10.1016/S1470-2045(14)70473-5 25439693

[6] Jiang Y, Li T, Liang X, . Association of adjuvant chemotherapy with survival in patients with stage II or III gastric cancer. JAMA Surg. 2017;152(7):e171087. doi:10.1001/jamasurg.2017.1087 28538950 PMC5831463

[7] Cox TR. The matrix in cancer. Nat Rev Cancer. 2021;21(4):217-238. doi:10.1038/s41568-020-00329-7 33589810

[8] Piersma B, Hayward MK, Weaver VM. Fibrosis and cancer: a strained relationship. Biochim Biophys Acta Rev Cancer. 2020;1873(2):188356. doi:10.1016/j.bbcan.2020.188356 32147542 PMC7733542

[9] Chen D, Liu Z, Liu W, . Predicting postoperative peritoneal metastasis in gastric cancer with serosal invasion using a collagen nomogram. Nat Commun. 2021;12(1):179. doi:10.1038/s41467-020-20429-0 33420057 PMC7794254

[10] Huang YL, Liang CY, Ritz D, . Collagen-rich omentum is a premetastatic niche for integrin α2-mediated peritoneal metastasis. Elife. 2020;9:e59442. doi:10.7554/eLife.59442 33026975 PMC7541088

[11] Chen D, Chen G, Jiang W, . Association of the collagen signature in the tumor microenvironment with lymph node metastasis in early gastric cancer. JAMA Surg. 2019;154(3):e185249. doi:10.1001/jamasurg.2018.5249 30698615 PMC6439641

[12] Provenzano PP, Eliceiri KW, Campbell JM, Inman DR, White JG, Keely PJ. Collagen reorganization at the tumor-stromal interface facilitates local invasion. BMC Med. 2006;4(1):38. doi:10.1186/1741-7015-4-38 17190588 PMC1781458

[13] Provenzano PP, Inman DR, Eliceiri KW, . Collagen density promotes mammary tumor initiation and progression. BMC Med. 2008;6:11. doi:10.1186/1741-7015-6-11 18442412 PMC2386807

[14] Han W, Chen S, Yuan W, . Oriented collagen fibers direct tumor cell intravasation. Proc Natl Acad Sci U S A. 2016;113(40):11208-11213. doi:10.1073/pnas.1610347113 27663743 PMC5056065

[15] Toss MS, Miligy IM, Gorringe KL, . Geometric characteristics of collagen have independent prognostic significance in breast ductal carcinoma in situ: an image analysis study. Mod Pathol. 2019;32(10):1473-1485. doi:10.1038/s41379-019-0296-7 31175326

[16] Hoover EE, Squier JA. Advances in multiphoton microscopy technology. Nat Photonics. 2013;7(2):93-101. doi:10.1038/nphoton.2012.361 24307915 PMC3846297

[17] Tu H, Liu Y, Turchinovich D, . Stain-free histopathology by programmable supercontinuum pulses. Nat Photonics. 2016;10(8):534-540. doi:10.1038/nphoton.2016.94 27668009 PMC5031149

[18] Campagnola P. Second harmonic generation imaging microscopy: applications to diseases diagnostics. Anal Chem. 2011;83(9):3224-3231. doi:10.1021/ac1032325 21446646 PMC3104727

[19] Chen X, Nadiarynkh O, Plotnikov S, Campagnola PJ. Second harmonic generation microscopy for quantitative analysis of collagen fibrillar structure. Nat Protoc. 2012;7(4):654-669. doi:10.1038/nprot.2012.009 22402635 PMC4337962

[20] Jiang Y, Zhang Q, Hu Y, . ImmunoScore signature: a prognostic and predictive tool in gastric cancer. Ann Surg. 2018;267(3):504-513. doi:10.1097/SLA.0000000000002116 28002059

[21] Zhang JX, Song W, Chen ZH, . Prognostic and predictive value of a microRNA signature in stage II colon cancer: a microRNA expression analysis. Lancet Oncol. 2013;14(13):1295-1306. doi:10.1016/S1470-2045(13)70491-1 24239208

[22] Tibshirani R. The lasso method for variable selection in the Cox model. Stat Med. 1997;16(4):385-395. doi:10.1002/(SICI)1097-0258(19970228)16:4<385::AID-SIM380>3.0.CO;2-3 9044528

[23] Tibshirani R. Regression shrinkage and selection via the lasso: a retrospective. J R Stat Soc B. 2011;73(3): 273-282. doi:10.1111/j.1467-9868.2011.00771.x

[24] Huang Y, Liu Z, He L, . Radiomics signature: a potential biomarker for the prediction of disease-free survival in early-stage (I or II) non-small cell lung cancer. Radiology. 2016;281(3):947-957. doi:10.1148/radiol.2016152234 27347764

[25] World Medical Association. World Medical Association Declaration of Helsinki: ethical principles for medical research involving human subjects. JAMA. 2013;310(20):2191-2194. doi:10.1001/jama.2013.281053 24141714

[26] In H, Solsky I, Palis B, . Validation of the 8th edition of the AJCC TNM staging system for gastric cancer using the National Cancer Database. *Ann Surg Oncol*. 2017;24(12):3683-3691. 10.1245/s10434-017-6078-x 28895113

[27] Barlow AM, Mostaço-Guidolin LB, Osei ET, Booth S, Hackett TL. Super resolution measurement of collagen fibers in biological samples: validation of a commercial solution for multiphoton microscopy. PLoS One. 2020;15(2):e0229278. doi:10.1371/journal.pone.0229278 32059025 PMC7021303

[28] Hothorn T, Zeileis A. Generalized maximally selected statistics. Biometrics. 2008;64(4):1263-1269. doi:10.1111/j.1541-0420.2008.00995.x 18325074

[29] Gerds TA, Kattan MW, Schumacher M, Yu C. Estimating a time-dependent concordance index for survival prediction models with covariate dependent censoring. Stat Med. 2013;32(13):2173-2184. doi:10.1002/sim.5681 23172755

[30] Kattan MW. Judging new markers by their ability to improve predictive accuracy. J Natl Cancer Inst. 2003;95(9):634-635. doi:10.1093/jnci/95.9.634 12734304

[31] Vickers AJ, Elkin EB. Decision curve analysis: a novel method for evaluating prediction models. Med Decis Making. 2006;26(6):565-574. doi:10.1177/0272989X06295361 17099194 PMC2577036

[32] Vickers AJ, Cronin AM, Elkin EB, Gonen M. Extensions to decision curve analysis, a novel method for evaluating diagnostic tests, prediction models and molecular markers. BMC Med Inform Decis Mak. 2008;8:53. doi:10.1186/1472-6947-8-53 19036144 PMC2611975

[33] Collins GS, Reitsma JB, Altman DG, Moons KG. Transparent Reporting of a Multivariable Prediction Model for Individual Prognosis Or Diagnosis (TRIPOD). Ann Intern Med. 2015;162(10):735-736. doi:10.7326/L15-5093-2 25984857

[34] Sun W, Chang S, Tai DC, . Nonlinear optical microscopy: use of second harmonic generation and two-photon microscopy for automated quantitative liver fibrosis studies. J Biomed Opt. 2008;13(6):064010. doi:10.1117/1.3041159 19123657

[35] Xu S, Kang CH, Gou X, . Quantification of liver fibrosis via second harmonic imaging of the Glisson’s capsule from liver surface. J Biophotonics. 2016;9(4):351-363. doi:10.1002/jbio.201500001 26131709 PMC5775478

[36] Ranstam J, Cook JA. LASSO regression. Br J Surg. 2018;105(10):1348. doi:10.1002/bjs.10895

[37] Welch DR, Hurst DR. Defining the hallmarks of metastasis. Cancer Res. 2019;79(12):3011-3027. doi:10.1158/0008-5472.CAN-19-0458 31053634 PMC6571042

[38] Hristu R, Eftimie LG, Stanciu SG, . Quantitative second harmonic generation microscopy for the structural characterization of capsular collagen in thyroid neoplasms. Biomed Opt Express. 2018;9(8):3923-3936. doi:10.1364/BOE.9.003923 30338165 PMC6191628

[39] Mostaço-Guidolin LB, Osei ET, Ullah J, . Defective fibrillar collagen organization by fibroblasts contributes to airway remodeling in asthma. Am J Respir Crit Care Med. 2019;200(4):431-443. doi:10.1164/rccm.201810-1855OC 30950644

[40] Paesen R, Smolders S, Vega JM, Eijnde BO, Hansen D, Ameloot M. Fully automated muscle quality assessment by Gabor filtering of second harmonic generation images. J Biomed Opt. 2016;21(2):26003. doi:10.1117/1.JBO.21.2.026003 26848544

[41] Westreich J, Khorasani M, Jones B, Demidov V, Nofech-Mozes S, Vitkin A. Novel methodology to image stromal tissue and assess its morphological features with polarized light: towards a tumour microenvironment prognostic signature. Biomed Opt Express. 2019;10(8):3963-3973. doi:10.1364/BOE.10.003963 31452988 PMC6701544

[42] Paget S. The distribution of secondary growths in cancer of the breast. 1889. Cancer Metastasis Rev. 1989;8(2):98-101. 2673568

[43] Gambardella V, Cervantes A. Precision medicine in the adjuvant treatment of gastric cancer. Lancet Oncol. 2018;19(5):583-584. doi:10.1016/S1470-2045(18)30131-1 29567072

[44] Xiong G, Stewart RL, Chen J, . Collagen prolyl 4-hydroxylase 1 is essential for HIF-1α stabilization and TNBC chemoresistance. Nat Commun. 2018;9(1):4456. doi:10.1038/s41467-018-06893-9 30367042 PMC6203834

[45] Chen J, Zhuo S, Chen G, . Establishing diagnostic features for identifying the mucosa and submucosa of normal and cancerous gastric tissues by multiphoton microscopy. Gastrointest Endosc. 2011;73(4):802-807. doi:10.1016/j.gie.2010.12.016 21457819

[46] He K, Zhao L, Huang X, . Label-free imaging for T staging of gastric carcinoma by multiphoton microscopy. Lasers Med Sci. 2018;33(4):871-882. doi:10.1007/s10103-018-2442-8 29411176

[47] Kakkad SM, Solaiyappan M, Argani P, . Collagen I fiber density increases in lymph node positive breast cancers: pilot study. J Biomed Opt. 2012;17(11):116017. doi:10.1117/1.JBO.17.11.116017 23117811 PMC3486274

