# Supplementary material for: Association of Tumor-Associated Collagen Signature With Prognosis and Adjuvant Chemotherapy Benefits in Patients With Gastric Cancer
Source: JAMA Netw Open. 2021 Nov 30;4(11):e2136388. doi: 10.1001/jamanetworkopen.2021.36388 (PMC8634059; doi:10.1001/jamanetworkopen.2021.36388)
Supplement: Supplement. — eMethods. Supplemental Methods eFigure 1. Study Design of This Study eFigure 2. Collagen Feature Selection Was Performed Using the LASSO-Cox Regression Model eFigure 3. The Optimal Cutoff Value for TACSGC Was Determined Using Maximally Selected Rank Statistics eFigure 4. Distribution of the TACSGC With Corresponding Survival Status in Training and Validation Cohorts eFigure 5. Kaplan-Meier Survival Analysis of DFS for All 519 Patients According to the TACSGC Level Stratified by Clinicopathological Variables eFigure 6. Kaplan-Meier Survival Analysis of OS for All 519 Patients According to the TACSGC Level Stratified by Clinicopathological Variables eFigure 7. Integrated Nomograms for DFS and OS eFigure 8. Calibration Curves of the Integrated Nomograms for DFS and OS eFigure 9. Time-Independent ROC Curves at 5 Years of Different Models for DFS and OS eFigure 10. Decision Curve Analysis of Each Model for Predicting Survival eFigure 11. Adjuvant Chemotherapy Benefits in Stage II GC Patients in Terms of DFS and OS eFigure 12. Adjuvant Chemotherapy Benefits in Stage III GC Patients in Terms of DFS and OS eTable 1. Summarization of the 146 Extracted Collagen Features eTable 2. Comparison of Clinicopathological Characteristics Between Patients With and Without Complete Data eTable 3. Clinicopathological Characteristics of Patients Grouped by TACSGC in the Training Cohort eTable 4. Clinicopathological Characteristics of Patients Grouped by TACSGC in the Validation Cohort eTable 5. Clinicopathological Characteristics of Patients Grouped by TACSGC in the Total Cohort eTable 6. Univariate Cox Regression Analyses of DFS and OS in Training Cohort eTable 7. Univariate Cox Regression Analyses of DFS and OS in Validation Cohort eTable 8. Multivariable Cox Regression Analyses of DFS and OS Without TACSGC eTable 9. C-index Comparison of Integrated Nomogram With Other Prediction Models eTable 10. The Net Reclassification Improvement of Adding the TACSGC to the Clinicopathological Model [file jamanetwopen-e2136388-s001.pdf]

Supplementary Online Content

Chen D, Chen H, Chi L, et al. Association of tumor-associated collagen signature with prognosis and adjuvant chemotherapy benefits in patients with gastric cancer. *JAMA Netw Open*. 2021;4(11):e2136388. doi:10.1001/jamanetworkopen.2021.36388

eMethods. Supplemental Methods
eFigure 1. Study Design of This Study
eFigure 2. Collagen Feature Selection Was Performed Using the LASSO-Cox Regression Model
eFigure 3. The Optimal Cutoff Value for $TACS_{GC}$ Was Determined Using Maximally Selected Rank Statistics
eFigure 4. Distribution of the $TACS_{GC}$ With Corresponding Survival Status in Training and Validation Cohorts
eFigure 5. Kaplan-Meier Survival Analysis of DFS for All 519 Patients According to the $TACS_{GC}$ Level Stratified by Clinicopathological Variables
eFigure 6. Kaplan-Meier Survival Analysis of OS for All 519 Patients According to the $TACS_{GC}$ Level Stratified by Clinicopathological Variables
eFigure 7. Integrated Nomograms for DFS and OS
eFigure 8. Calibration Curves of the Integrated Nomograms for DFS and OS
eFigure 9. Time-Independent ROC Curves at 5 Years of Different Models for DFS and OS
eFigure 10. Decision Curve Analysis of Each Model for Predicting Survival
eFigure 11. Adjuvant Chemotherapy Benefits in Stage II GC Patients in Terms of DFS and OS
eFigure 12. Adjuvant Chemotherapy Benefits in Stage III GC Patients in Terms of DFS and OS
eTable 1. Summarization of the 146 Extracted Collagen Features
eTable 2. Comparison of Clinicopathological Characteristics Between Patients With and Without Complete Data
eTable 3. Clinicopathological Characteristics of Patients Grouped by $TACS_{GC}$ in the Training Cohort
eTable 4. Clinicopathological Characteristics of Patients Grouped by $TACS_{GC}$ in the Validation Cohort
eTable 5. Clinicopathological Characteristics of Patients Grouped by $TACS_{GC}$ in the Total Cohort
eTable 6. Univariate Cox Regression Analyses of DFS and OS in Training Cohort
eTable 7. Univariate Cox Regression Analyses of DFS and OS in Validation Cohort
eTable 8. Multivariable Cox Regression Analyses of DFS and OS Without $TACS_{GC}$
eTable 9. C-index Comparison of Integrated Nomogram With Other Prediction Models
eTable 10. The Net Reclassification Improvement of Adding the $TACS_{GC}$ to the Clinicopathological Model
eTable 11. Clinicopathological Characteristics of Stage II and III Patients According to Chemotherapy
eTable 12. Adjuvant Chemotherapy Interaction With the $TACS_{GC}$ for DFS and OS in Stage II and III Patients
eReferences

This supplementary material has been provided by the authors to give readers additional information about their work.

© 2021 Chen D et al. *JAMA Network Open.*

eMethods. Supplemental Methods

1. Multiphoton imaging system

The commercial laser scanning multiphoton microscope platform (LSM 880 Zeiss, Germany) was equipped with a mode-locked femtosecond Ti:sapphire laser with tunable wavelengths from 690 to 1064 nm (Chameleon Ultra, Coherent, USA). The excitation wavelength (λ_{ex}) used in this study was 800 nm. A Plan-Apochromat 20× objective (Zeiss, Germany) was employed for focusing the excitation beam and for collecting the backward signals. The two-channel mode achieved two-photon excitation fluorescence (TPEF) and second harmonic generation (SHG) signals, which were separated by a dichroic mirror in the detection path. One channel corresponds to a wavelength range of 430 to 708 nm to show the morphologies of the tissue components from the TPEF signals (red color), whereas the other channel covers the wavelength range from 387 to 409 nm to present the microstructures of the tissue components from the SHG signals (green color).

2. Collagen feature extraction

For the extraction of morphological features, the Gaussian mixture model method was first used to segment the SHG image into collagen pixels and background pixels,[1] which was named the binary collagen mask image. Then, the binary collagen mask image was processed using a fiber network extraction method to trace each collagen fiber in the image and determine the cross-link points.[2] A cross-link point was defined as the connecting point between two or more fibers. Meanwhile, an orientation index was quantified to represent the collagen alignment using the Fourier transform spectra method.[3] For the extraction of histogram-based features, a histogram-based approach was used. The mean, variation, skewness, kurtosis, energy and entropy were calculated from the histogram of the SHG pixel intensity distribution. Moreover, the contrast, correlation, energy and homogeneity were calculated from the gray-level concurrence matrix (GLCM) of SHG images with five different displacements of pixels at 1, 2, 3, 4 and 5 and four different directions at 0, 45, 90 and 135 degrees. In addition, the SHG images were convolved with Gabor filters at four different scales and six different orientations to extract the Gabor wavelet transform features, and the mean and variation in the magnitude of the convolution over the image at each setting were calculated.[4]

© 2021 Chen D et al. *JAMA Network Open.*

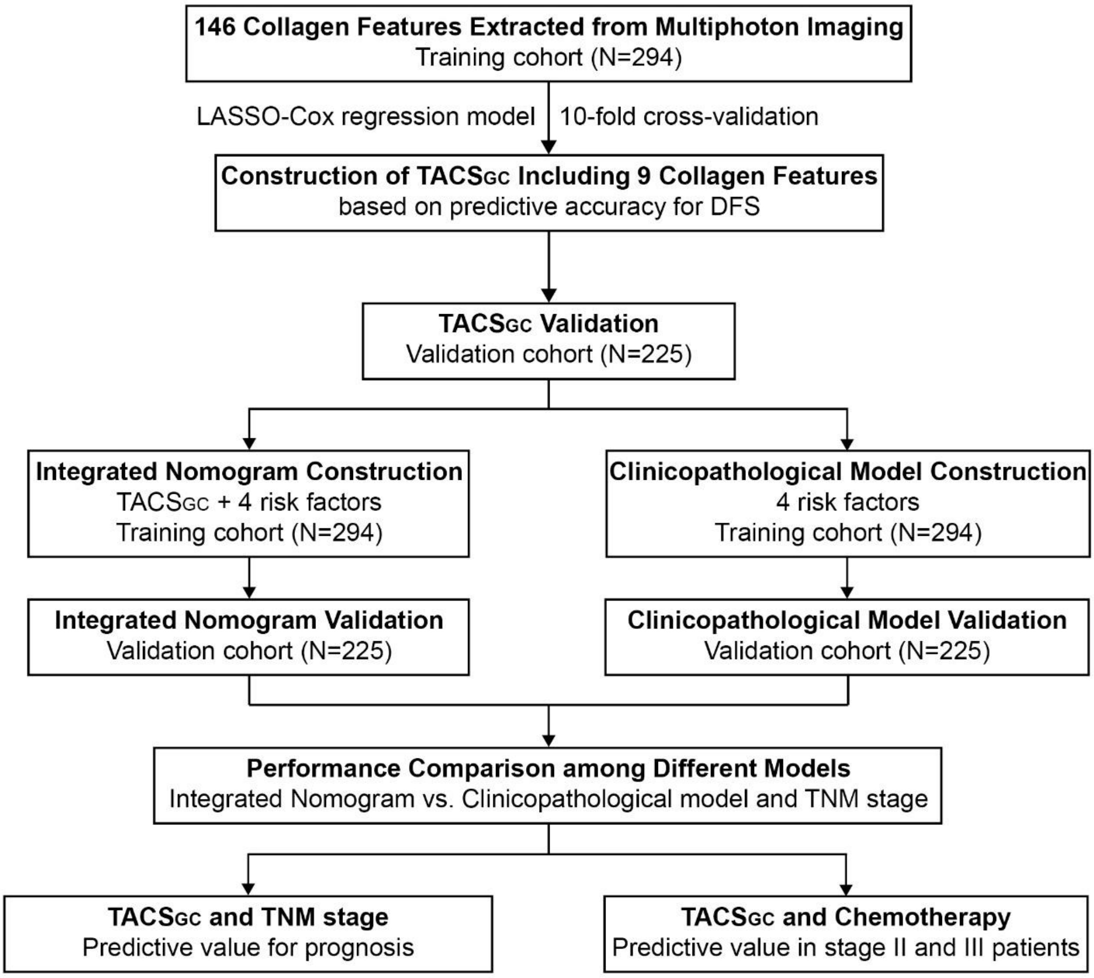

eFigure 1. Study design of this study.

Abbreviation: $TACS_{GC}$, tumor-associated collagen signature of gastric cancer; DFS, disease-free survival; TNM, tumor-node-metastasis.

© 2021 Chen D et al. *JAMA Network Open.*

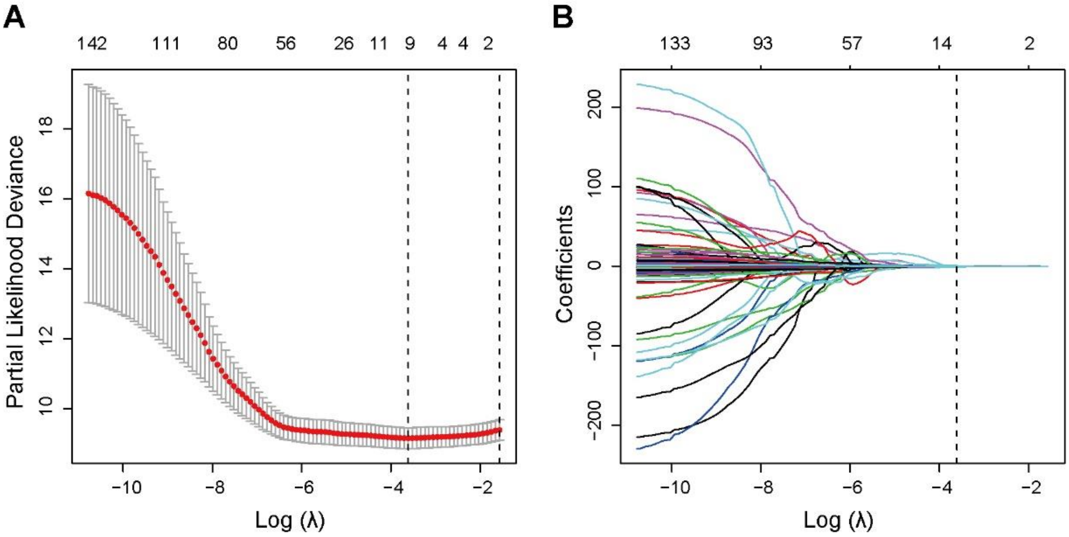

eFigure 2. Collagen feature selection was performed using the LASSO-Cox regression model.

(A) Penalty parameter (λ) selection in the LASSO-Cox regression method using 10-fold cross-validation via minimum partial likelihood deviance. Solid vertical lines represent the partial likelihood deviance ± SE. Dashed vertical lines were drawn at the optimal values by the minimum criteria (left) and the 1-SE criteria (right). A λ value of 0.027, with log (λ) = -3.611, was chosen by 10-fold cross-validation via minimum criteria. (B) LASSO coefficient profiles of the 146 collagen features. A dashed vertical line was drawn at the value of log (λ) = -3.611 using 10-fold cross-validation, wherein the optimal λ resulted in 9 nonzero coefficients. *Abbreviation:* $TACS_{GC}$: tumor-associated collagen signature of gastric cancer; LASSO: least absolute shrinkage and selection operator; SE: standard error.

© 2021 Chen D et al. *JAMA Network Open.*

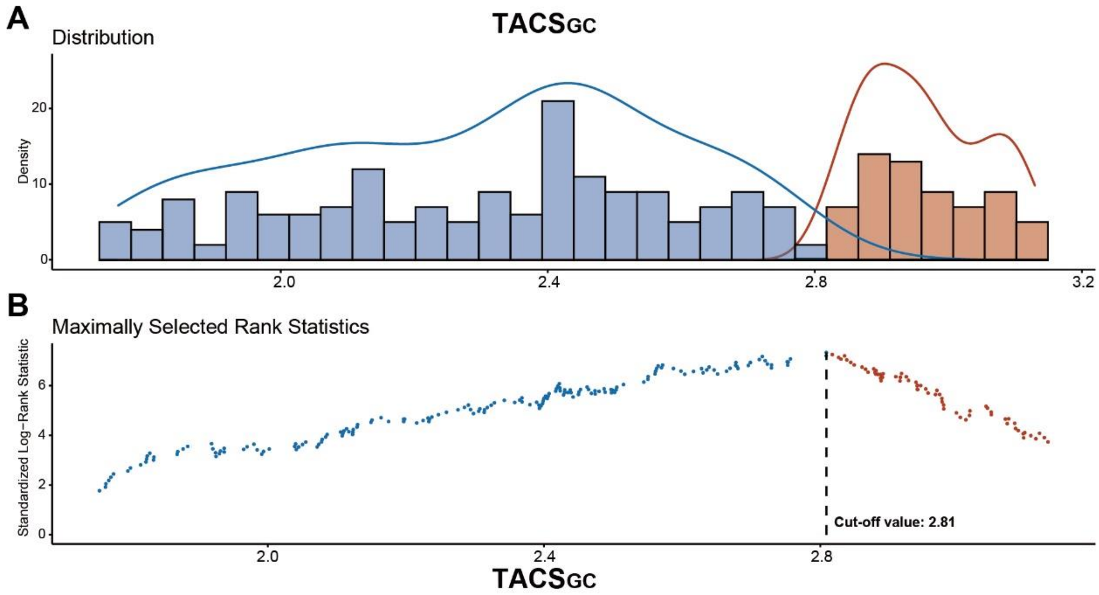

eFigure 3. The optimal cutoff value for $\mathrm{TACS_{GC}}$ was determined using maximally selected rank statistics.

(A) Histogram shows the density distribution for the high- and low-$\mathrm{TACS_{GC}}$ groups divided by the optimal cutoff value. (B) Scatter plot shows the standardized log-rank statistics for each corresponding $\mathrm{TACS_{GC}}$ cutoff value. The optimal cutoff value with the maximum standardized log-rank statistic is marked with a vertical dashed line. *Abbreviation:* $\mathrm{TACS_{GC}}$, tumor-associated collagen signature of gastric cancer.

© 2021 Chen D et al. *JAMA Network Open.*

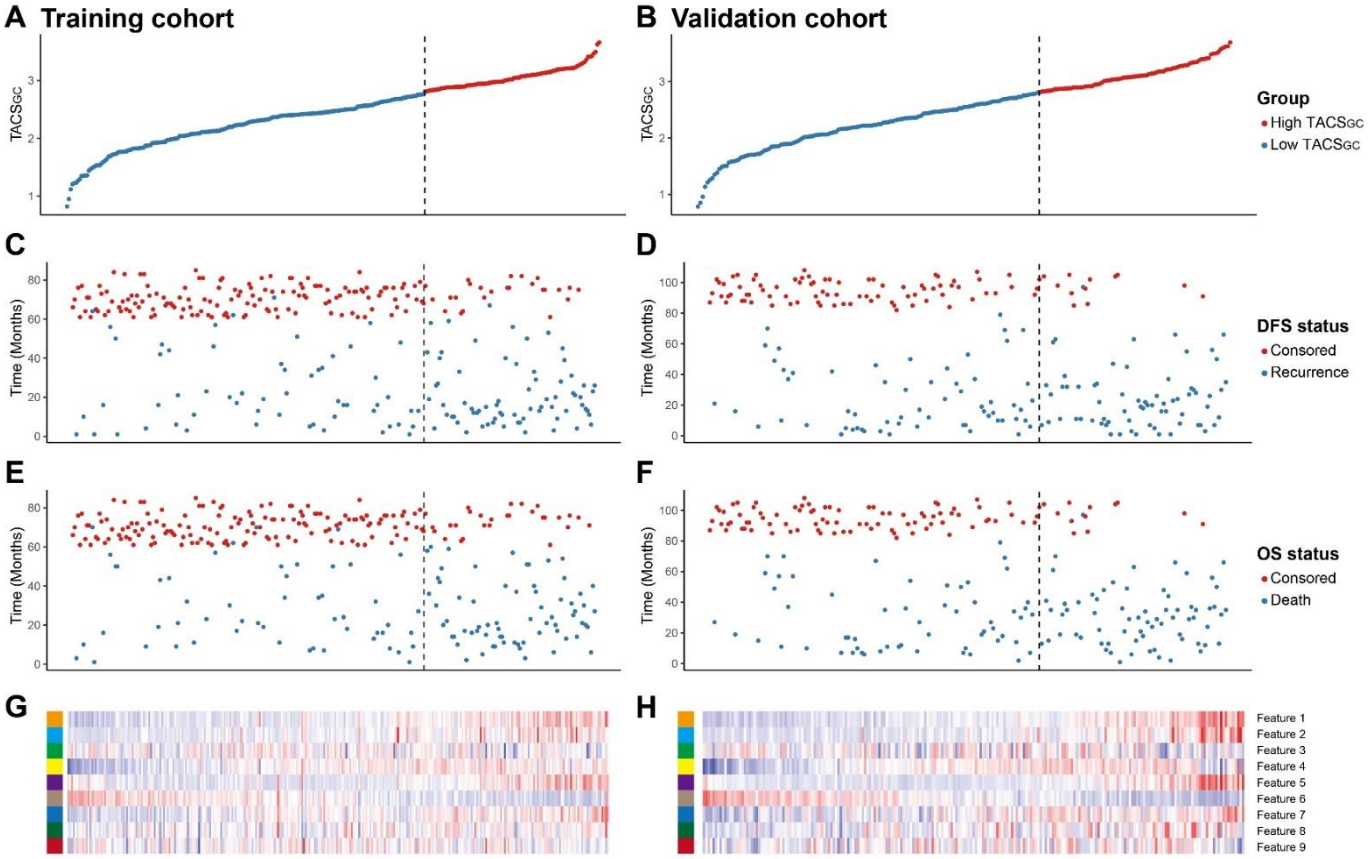

eFigure 4. Distribution of TACS_{GC} with corresponding survival status in the training and validation cohorts.

(A-B) TACS_{GC} distribution. (C-D) DFS status of GC patients. (E-F) OS status of GC patients. (G-H) Color gram of the expression profiles of nine collagen features in GC patients; rows represent nine collagen features, and columns represent patients. The vertical dashed line represents the cutoff value of the TACS_{GC} that was used to divide the patients into high- and low-TACS_{GC} groups. *Abbreviation:* TACS_{GC}, tumor-associated collagen signature of gastric cancer; GC, gastric cancer; DFS, disease-free survival; OS, overall survival.

© 2021 Chen D et al. *JAMA Network Open.*

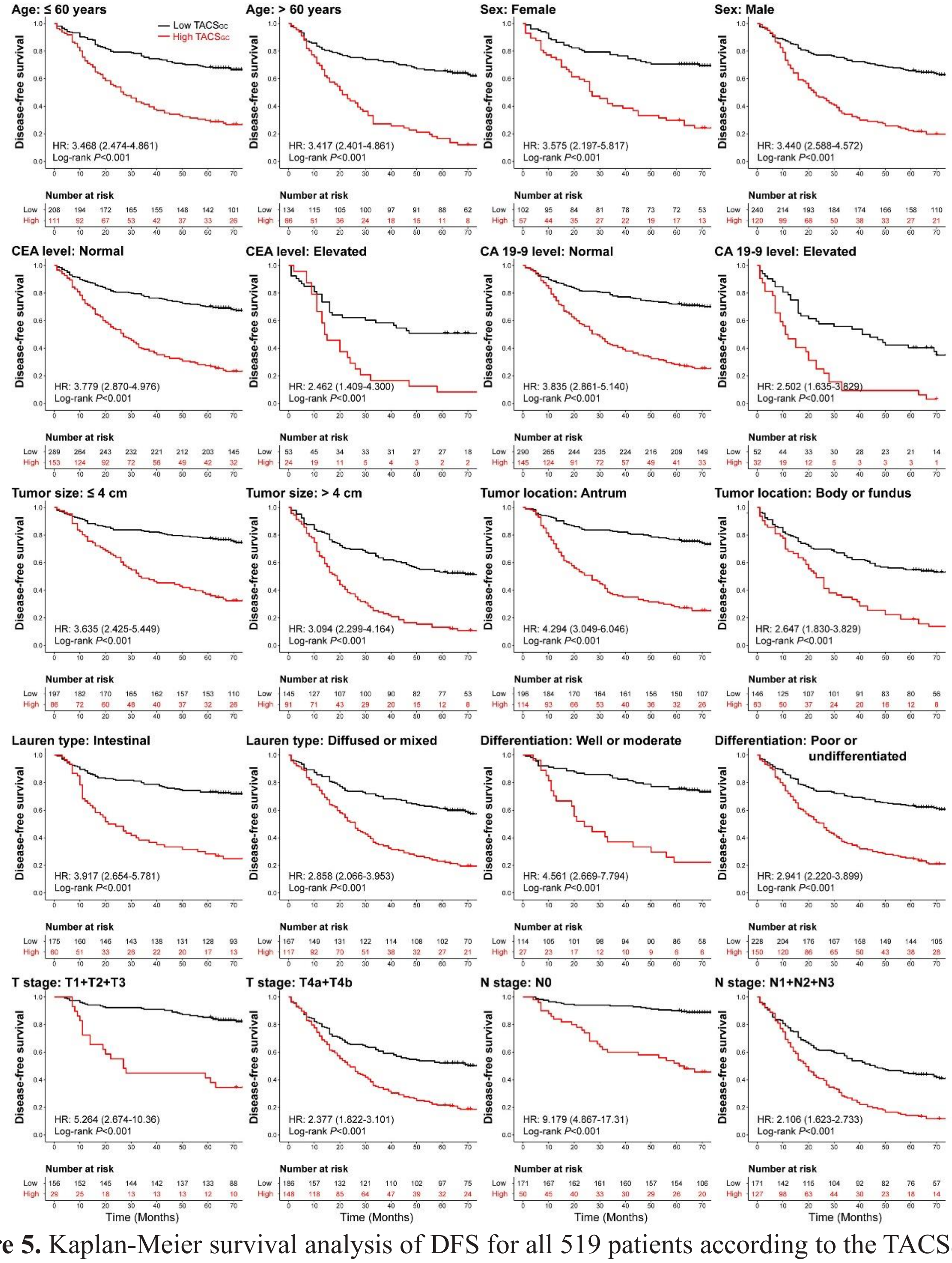

eFigure 5. Kaplan-Meier survival analysis of DFS for all 519 patients according to the $TACS_{GC}$ level stratified by clinicopathological variables.

Abbreviation: $TACS_{GC}$, tumor-associated collagen signature of gastric cancer; CEA, carcinoembryonic antigen; CA, cancer antigen; DFS, disease-free survival.

© 2021 Chen D et al. *JAMA Network Open.*

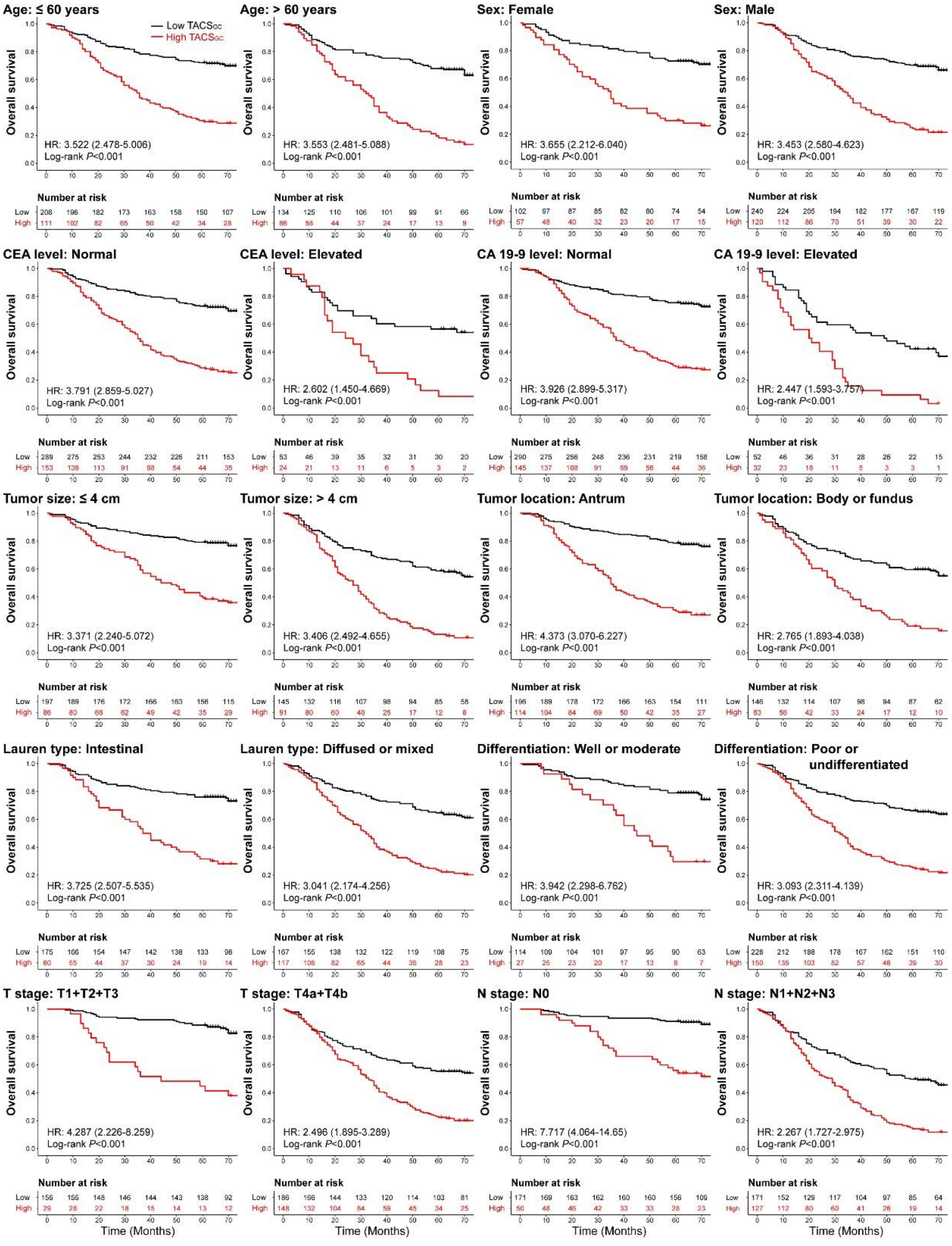

eFigure 6. Kaplan-Meier survival analysis of OS for all 519 patients according to the $TACS_{GC}$ level stratified by clinicopathological variables.

Abbreviation: $TACS_{GC}$, tumor-associated collagen signature of gastric cancer; CEA, carcinoembryonic antigen; CA, cancer antigen; OS, overall survival.

© 2021 Chen D et al. *JAMA Network Open.*

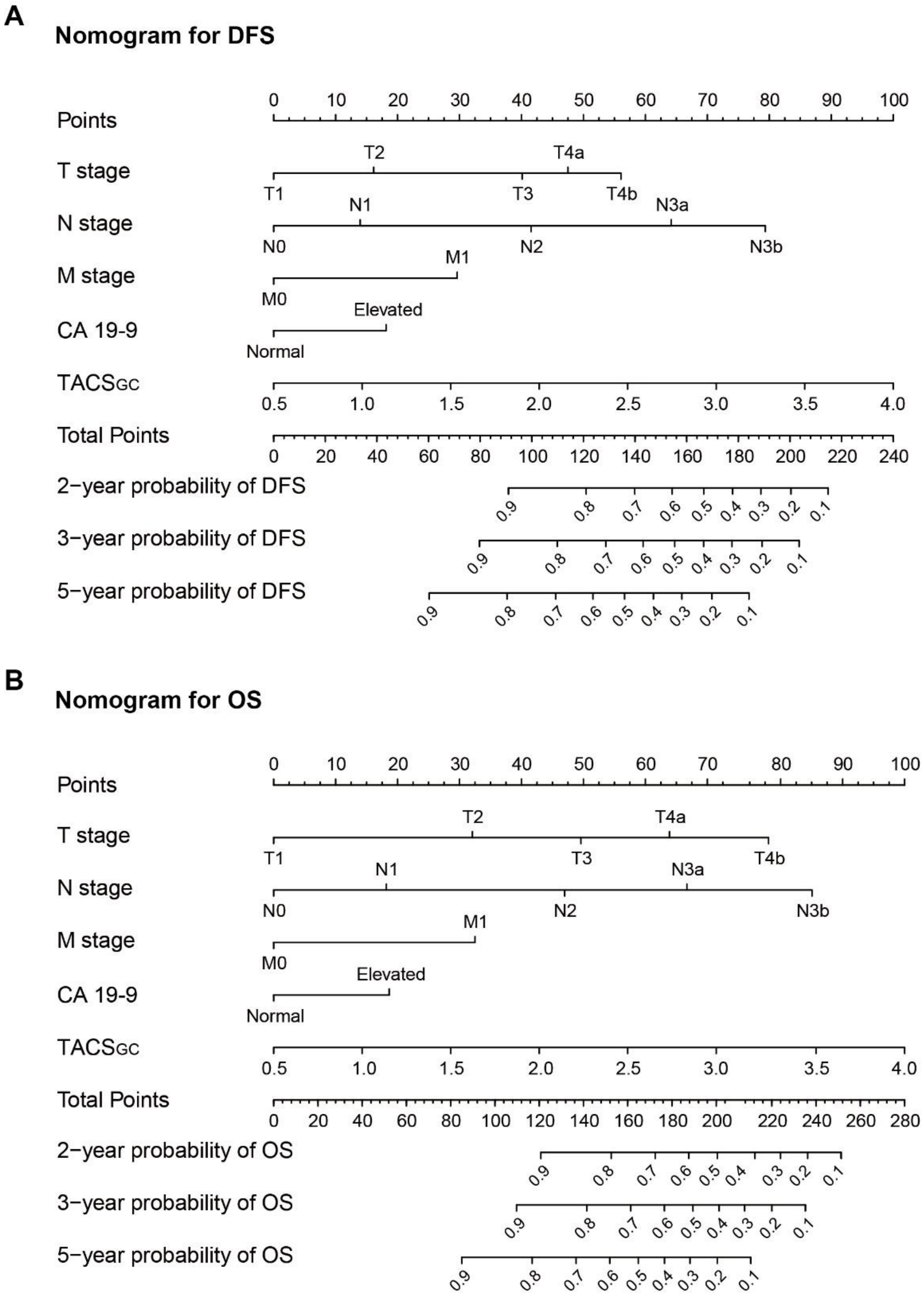

eFigure 7. Integrated nomograms for DFS and OS.

(A) Integrated nomogram for DFS. (B) Integrated nomogram for OS. To use the nomogram, first locate the patient's T stage on the T stage axis. Then, draw a line straight upward to the Points axis to determine how many points the patient receives for T stage. Repeat this process for each variable, and sum the points obtained from each risk factor. Finally, locate the final sum on the Total point axis. Draw a line straight down to find the patient's probability of survival.

Abbreviation: DFS, disease-free survival; OS, overall survival; CA, cancer antigen; $TACS_{GC}$, tumor-associated collagen signature of gastric cancer.

© 2021 Chen D et al. *JAMA Network Open.*

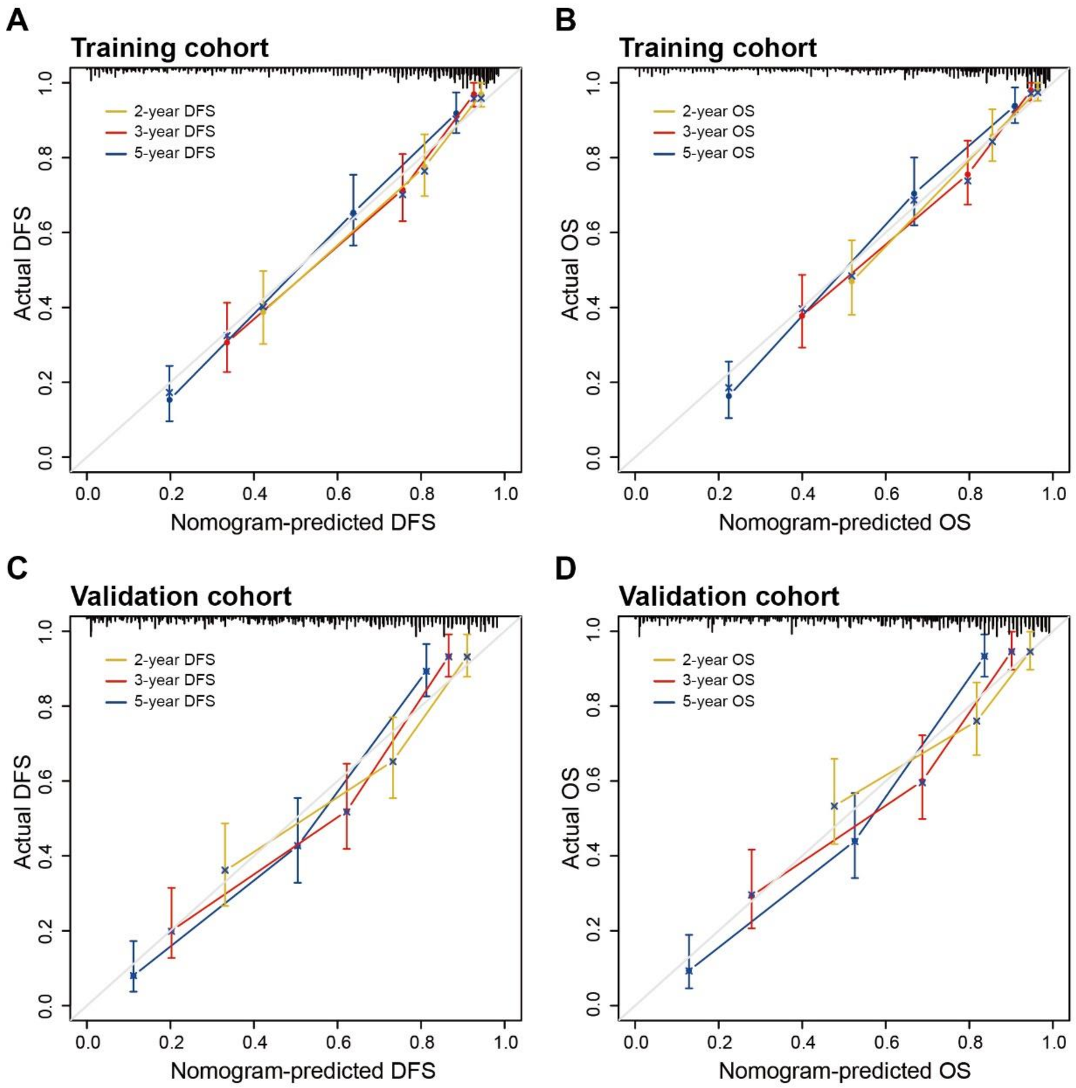

eFigure 8. Calibration curves of the integrated nomograms for DFS and OS.

(A) Calibration curves at 2 years, 3 years, and 5 years of the integrated nomogram for DFS in the training cohort. (B) Calibration curves at 2 years, 3 years, and 5 years of the integrated nomogram for OS in the training cohort. (C) Calibration curves at 2 years, 3 years, and 5 years of the integrated nomogram for DFS in the validation cohort. (D) Calibration curves at 2 years, 3 years, and 5 years of the integrated nomogram for OS in the validation cohort. The nomogram-predicted survival is plotted on the *x*-axis; the actual likelihood of survival is plotted on the *y*-axis. The calibration curve shows the calibration of the integrated nomograms in terms of the agreement between the nomogram-predicted survival and the actual likelihood of survival. The diagonal gray line represents a perfect prediction by an ideal nomogram, and other solid lines indicate the performance of the nomogram. A closer alignment with the diagonal line represents a better estimation. *Abbreviation:* DFS, disease-free survival; OS, overall survival.

© 2021 Chen D et al. *JAMA Network Open.*

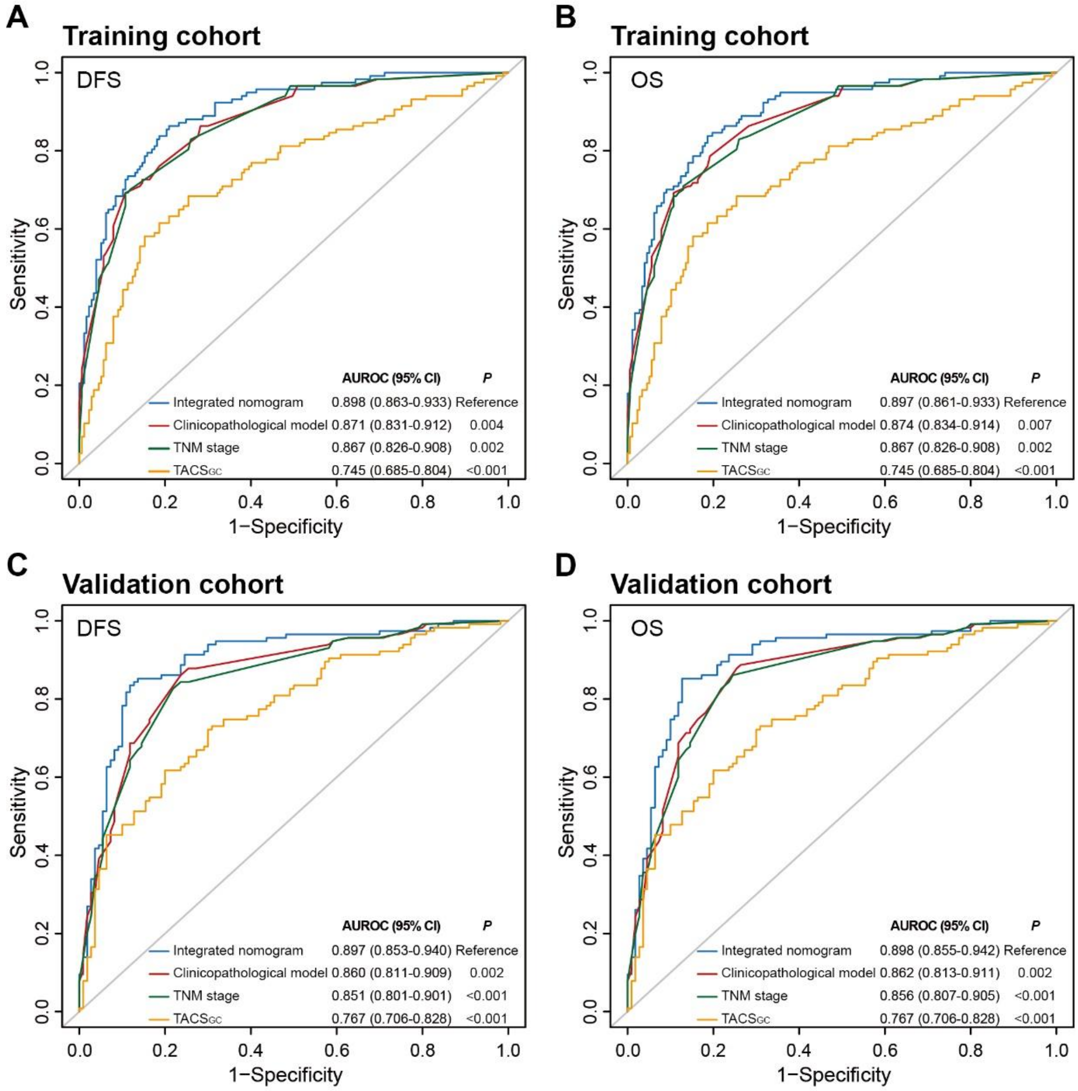

eFigure 9. Time-independent ROC curves at 5 years of different models for DFS and OS.

(A) Time-independent ROC curves of different models for DFS in the training cohort. (B) Time-independent ROC curves of different models for OS in the training cohort. (C) Time-independent ROC curves of different models for DFS in the validation cohort. (D) Time-independent ROC curves of different models for OS in the validation cohort. *Abbreviation:* DFS, disease-free survival; OS, overall survival; $TACS_{GC}$, tumor-associated collagen signature of gastric cancer; TNM, tumor-node-metastasis; AUROC, area under the receiver operator characteristic curve; ROC, receiver operator characteristic.

© 2021 Chen D et al. *JAMA Network Open.*

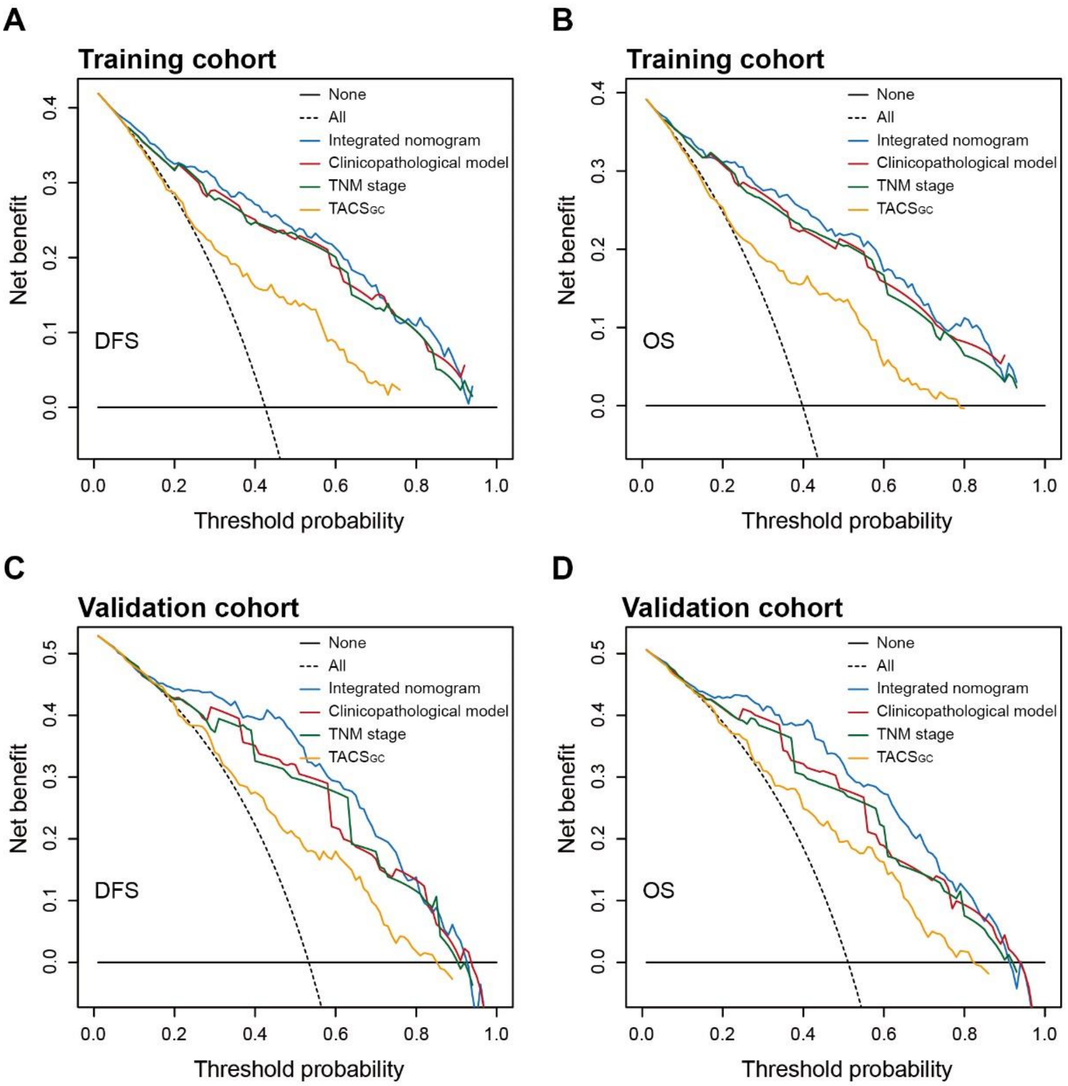

eFigure 10. Decision curve analysis of each model for predicting survival.

(A) Decision curve analysis of each model of DFS in the training cohort. (B) Decision curve analysis of each model for OS in the training cohort. (C) Decision curve analysis of each model for DFS in the validation cohort. (D) Decision curve analysis of each model for OS in the validation cohort. The *y*-axis measures the net benefit. The net benefit is calculated by summing the benefits (true positive results) and subtracting the harms (false positive results), weighting the latter by a factor related to the relative harm of an undetected cancer compared with the harm of unnecessary treatment. The integrated nomograms have the highest net benefit compared to other models across the full range of threshold probabilities. *Abbreviation:* DFS, disease-free survival; OS, overall survival; $TACS_{GC}$, tumor-associated collagen signature of gastric cancer; TNM, tumor-node-metastasis.

© 2021 Chen D et al. *JAMA Network Open.*

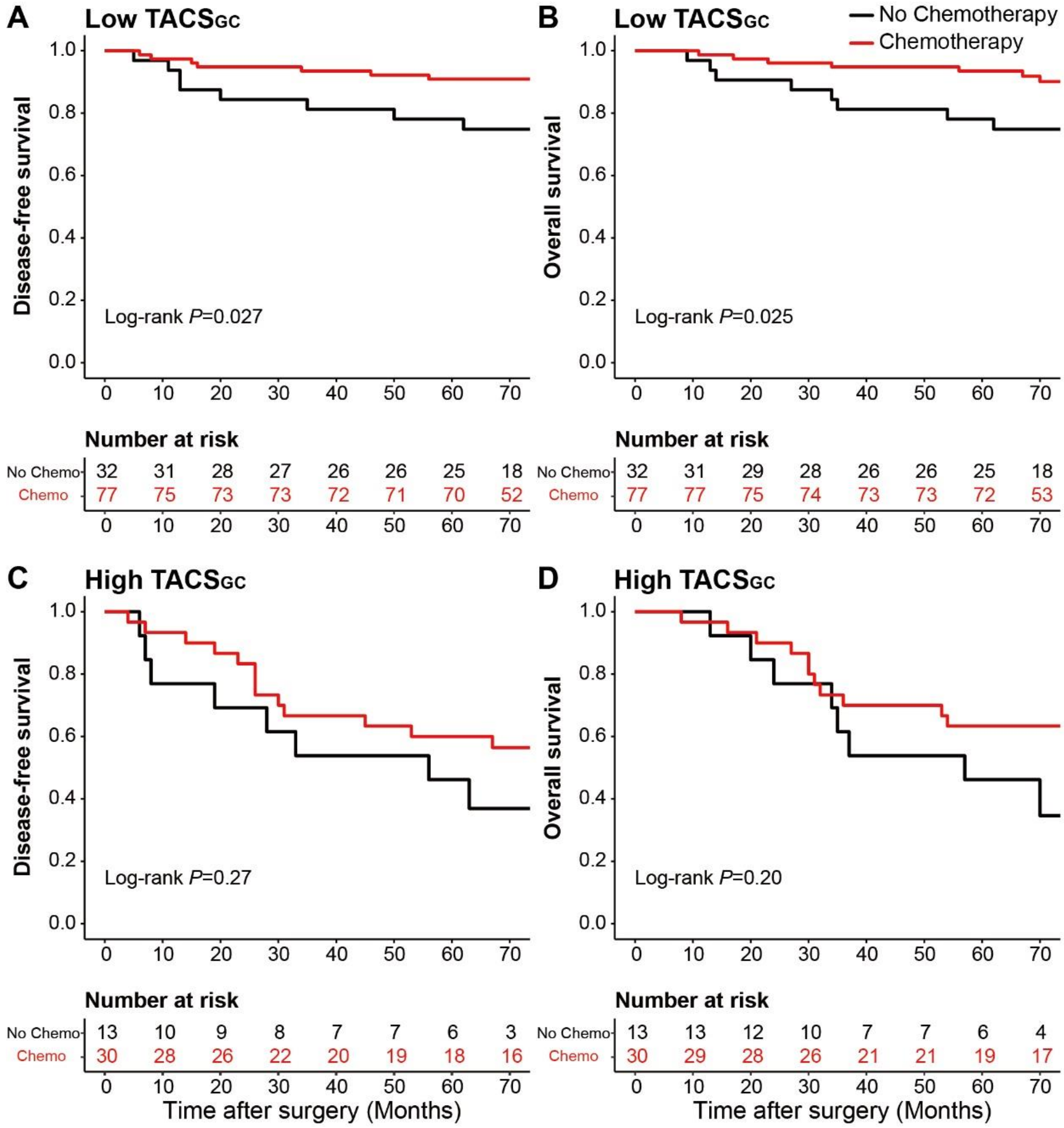

eFigure 11. Adjuvant chemotherapy benefits in stage III C patients in terms of DFS and OS.

(A) DFS comparison according to the receipt of adjuvant chemotherapy in low-$TACS_{GC}$ stage II GC patients. (B) OS comparison according to the receipt of adjuvant chemotherapy in low-$TACS_{GC}$ stage II GC patients. (C) DFS comparison according to the receipt of adjuvant chemotherapy in high-$TACS_{GC}$ stage II GC patients. (D) OS comparison according to the receipt of adjuvant chemotherapy in high-$TACS_{GC}$ stage II GC patients. *Abbreviation:* $TACS_{GC}$, tumor-associated collagen signature of gastric cancer; GC, gastric cancer; DFS, disease-free survival; OS, overall survival; Chemo, chemotherapy.

© 2021 Chen D et al. *JAMA Network Open.*

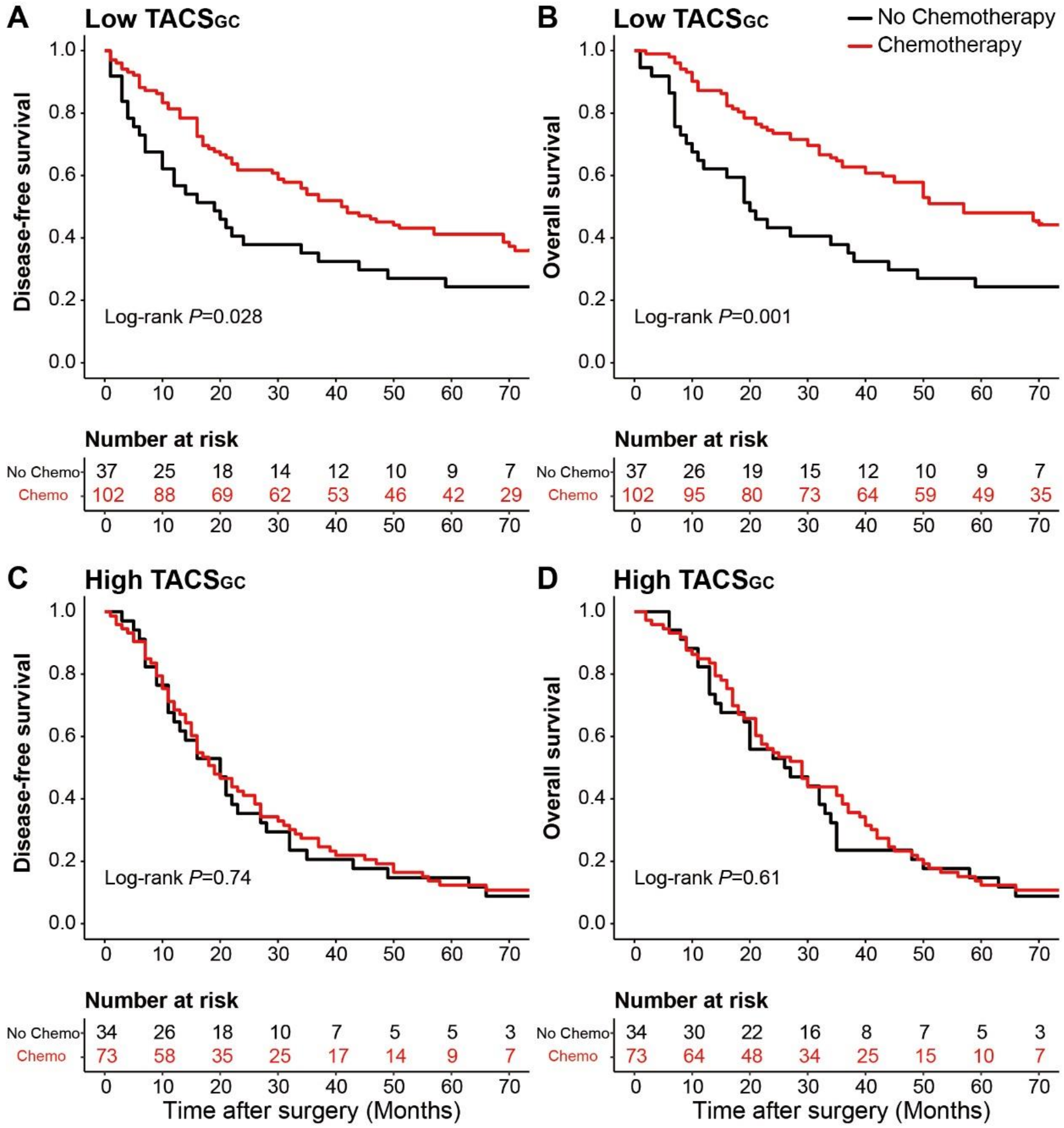

eFigure 12. Adjuvant chemotherapy benefits in stage II GC patients in terms of DFS and OS.

(A) DFS comparison according to the receipt of adjuvant chemotherapy in low-$TACS_{GC}$ stage III GC patients. (B) OS comparison according to the receipt of adjuvant chemotherapy in low-$TACS_{GC}$ stage III GC patients. (C) DFS comparison according to the receipt of adjuvant chemotherapy in high-$TACS_{GC}$ stage III GC patients. (D) OS comparison according to the receipt of adjuvant chemotherapy in high-$TACS_{GC}$ stage III GC patients. *Abbreviation:* $TACS_{GC}$, tumor-associated collagen signature of gastric cancer; GC, gastric cancer; DFS, disease-free survival; OS, overall survival; Chemo, chemotherapy.

© 2021 Chen D et al. *JAMA Network Open.*

eTable 1. Summarization of the 146 extracted collagen features

No.	Feature descriptions
**Morphological features**	
1-2	Mean and variation of collagen area
3-4	Mean and variation of collagen length
5-6	Mean and variation of collagen width
7-8	Mean and variation of collagen straightness
9-10	Mean and variation of collagen cross-link density
11-12	Mean and variation of collagen orientation
**Textural features**	
Histogram-based features	
13-18	Mean, variation, skewness, kurtosis, energy, and entropy
GLCM-based features	
19-98	Contrast, correlation, energy and homogeneity from the GLCM given five different pixel distances with four different directions
Gabor wavelet transform features	
99-146	Mean and variation in the convolution over the image with the Gabor filter at four scales with six orientations

Abbreviation: GLCM, gray-level co-occurrence matrix.

© 2021 Chen D et al. *JAMA Network Open.*

eTable 2. Comparison of clinicopathological characteristics between patients with and without complete data

Variables	Complete data group (n=519)	Incomplete data group (n=31)	*P*
**Age, No. (%)**			
≤ 60 years	319 (61.5)	21 (67.7)	0.49
> 60 years	200 (38.5)	10 (32.3)	
**Age[a], median (IQR), y**	57 (49-65)	56 (44-62)	0.17
**Sex, No. (%)**			
Male	360 (69.4)	22 (71.0)	0.85
Female	159 (30.6)	9 (29.0)	
**Tumor location, No. (%)**			
Fundus of stomach	113 (21.8)	8 (25.8)	0.82
Body of stomach	96 (18.5)	5 (16.1)	
Antrum of stomach	310 (59.7)	22 (58.0)	
**Tumor differentiation, No. (%)**			
Well	34 (6.6)	4 (12.9)	0.36
Moderate	107 (20.6)	7 (22.6)	
Poor and undifferentiated	378 (72.8)	20 (64.5)	
**Depth of invasion, No. (%)**			
T1	91 (17.6)	7 (22.6)	0.71
T2	46 (8.9)	4 (12.9)	
T3	48 (9.2)	4 (12.9)	
T4a	286 (55.1)	14 (45.2)	
T4b	48 (9.2)	2 (6.4)	
**Lymph node metastasis, No. (%)**			
N0	221 (42.5)	18 (58.1)	0.23
N1	96 (18.5)	6 (19.3)	
N2	85 (16.2)	5 (16.1)	
N3a	71 (13.6)	2 (6.5)	
N3b	48 (9.2)	0 (0.0)	
**Distant metastasis, No. (%)**			
M0	499 (96.1)	30 (96.8)	>0.99
M1	20 (3.9)	1 (3.2)	

Abbreviation: IQR, interquartile range.

[a] Median difference of age between with and without complete data: 3 (95% confidence interval: -1 to 7) years.

© 2021 Chen D et al. *JAMA Network Open.*

eTable 3. Clinicopathological characteristics of patients grouped by $TACS_{GC}$ in the training cohort

Variables	High $TACS_{GC}$ (n=96)	Low $TACS_{GC}$ (n=198)	*P*
**Age, No. (%)**			
≤ 60 years	62 (64.6)	123 (62.1)	0.68
> 60 years	34 (35.4)	75 (37.9)	
**Age[a], median (IQR), y**	56 (46.25-65)	58 (49-64)	0.29
**Sex, No. (%)**			
Male	69 (71.9)	139 (70.2)	0.77
Female	27 (28.1)	59 (29.8)	
**CEA level[b], No. (%)**			
Normal	82 (85.4)	163 (82.3)	0.51
Elevated	14 (14.6)	35 (17.7)	
**CA 19-9 level[c], No. (%)**			
Normal	79 (82.3)	171 (86.4)	0.36
Elevated	17 (17.7)	27 (17.7)	
**Tumor location, No. (%)**			
Fundus of stomach	22 (22.9)	48 (24.2)	0.97
Body of stomach	17 (17.7)	34 (17.2)	
Antrum of stomach	57 (59.4)	116 (58.6)	
**Tumor size, No. (%)**			
≤ 4 cm	49 (51.0)	121 (61.1)	0.11
> 4 cm	47 (49.0)	77 (38.9)	
**Tumor differentiation, No. (%)**			
Well	1 (1.0)	19 (9.6)	0.008
Moderate	17 (17.7)	46 (23.2)	
Poor and undifferentiated	78 (81.3)	133 (67.2)	
**Lauren type, No. (%)**			
Intestinal type	39 (40.6)	98 (49.5)	0.15
Diffused or mixed type	57 (59.4)	100 (50.5)	
**Depth of invasion, No. (%)**			
T1	4 (4.2)	57 (28.8)	<0.001
T2	5 (5.2)	21 (10.6)	
T3	5 (5.2)	25 (12.6)	
T4a	72 (75.0)	83 (41.9)	
T4b	10 (10.4)	12 (6.1)	
**Lymph node metastasis, No. (%)**			
N0	31 (32.3)	104 (52.4)	<0.001
N1	19 (19.8)	33 (16.7)	
N2	11 (11.5)	33 (16.7)	
N3a	25 (26.0)	14 (7.1)	
N3b	10 (10.4)	14 (7.1)	

© 2021 Chen D et al. *JAMA Network Open.*

**Distant metastasis, No. (%)**			
M0	86 (89.6)	197 (99.5)	<0.001
M1	10 (10.4)	1 (0.5)	
**TNM stage, No. (%)**			
Stage I	6 (6.2)	63 (31.8)	<0.001
Stage II	23 (24.0)	59 (29.8)	
Stage III	57 (59.4)	75 (37.9)	
Stage IV	10 (10.4)	1 (0.5)	

Abbreviation: $TACS_{GC}$, tumor-associated collagen signature of gastric cancer; IQR, interquartile range; CEA: carcinoembryonic antigen; CA: cancer antigen; TNM, tumor-node-metastasis.

SI conversion factor: To convert CEA to micrograms per liter, multiply by 1.0.

[a] Median difference of age between high- and low-$TACS_{GC}$: 2 (95% confidence interval: -1 to 5) years.

[b] For CEA, elevated indicates 5 ng/mL or greater; normal, less than 5 ng/mL.

[c] For CA 19-9, elevated indicates 37 U/mL or greater; normal, less than 37 U/mL.

© 2021 Chen D et al. *JAMA Network Open.*

eTable 4. Clinicopathological characteristics of patients grouped by $TACS_{GC}$ in the validation cohort

Variables	High $TACS_{GC}$ (n=81)	Low $TACS_{GC}$ (n=144)	*P*
**Age, No. (%)**			
≤ 60 years	49 (60.5)	85 (59.0)	0.83
> 60 years	32 (39.5)	59 (41.0)	
**Age[a], median (IQR), y**	57 (51.5-64.5)	58.5 (51-65)	0.81
**Sex, No. (%)**			
Male	51 (63.0)	101 (70.1)	0.27
Female	30 (37.0)	43 (29.9)	
**CEA level[b], No. (%)**			
Normal	82 (85.4)	163 (82.3)	0.97
Elevated	14 (14.6)	35 (17.7)	
**CA 19-9 level[c], No. (%)**			
Normal	65 (80.2)	120 (83.3)	0.56
Elevated	16 (19.8)	24 (16.7)	
**Tumor location, No. (%)**			
Fundus of stomach	10 (12.3)	33 (22.9)	0.07
Body of stomach	14 (17.3)	31 (21.5)	
Antrum of stomach	57 (70.4)	80 (55.6)	
**Tumor size, No. (%)**			
≤ 4 cm	37 (45.7)	76 (52.8)	0.31
> 4 cm	44 (54.3)	68 (47.2)	
**Tumor differentiation, No. (%)**			
Well	3 (3.7)	11 (7.6)	0.001
Moderate	6 (7.4)	38 (26.4)	
Poor and undifferentiated	72 (88.9)	95 (66.0)	
**Lauren type, No. (%)**			
Intestinal type	21 (25.9)	77 (53.5)	<0.001
Diffused or mixed type	60 (74.1)	67 (46.5)	
**Depth of invasion, No. (%)**			
T1	4 (4.9)	26 (18.1)	0.02
T2	4 (4.9)	16 (11.1)	
T3	7 (8.6)	11 (7.6)	
T4a	57 (70.4)	74 (51.4)	
T4b	9 (11.2)	17 (11.8)	
**Lymph node metastasis, No. (%)**			
N0	19 (23.5)	67 (46.5)	0.02
N1	18 (22.2)	24 (16.7)	
N2	20 (24.7)	21 (14.6)	
N3a	14 (17.3)	18 (12.5)	
N3b	10 (12.3)	14 (9.7)	

© 2021 Chen D et al. *JAMA Network Open.*

**Distant metastasis, No. (%)**			
M0	73 (90.1)	143 (99.3)	0.001
M1	8 (9.9)	1 (0.7)	
**TNM stage, No. (%)**			
Stage I	3 (3.7)	29 (20.1)	<0.001
Stage II	20 (24.7)	50 (34.7)	
Stage III	51 (63.0)	63 (43.8)	
Stage IV	7 (8.6)	2 (1.4)	

Abbreviation: $TACS_{GC}$, tumor-associated collagen signature of gastric cancer; IQR, interquartile range; CEA: carcinoembryonic antigen; CA: cancer antigen; TNM, tumor-node-metastasis.

SI conversion factor: To convert CEA to micrograms per liter, multiply by 1.0.

[a] Median difference of age between high- and low-$TACS_{GC}$: 0 (95% confidence interval: -3 to 4) year.

[b] For CEA, elevated indicates 5 ng/mL or greater; normal, less than 5 ng/mL.

[c] For CA 19-9, elevated indicates 37 U/mL or greater; normal, less than 37 U/mL.

© 2021 Chen D et al. *JAMA Network Open.*

eTable 5. Clinicopathological characteristics of patients grouped by $TACS_{GC}$ in the total cohort

Variables	High $TACS_{GC}$ (n=177)	Low $TACS_{GC}$ (n=342)	*P*
**Age, No. (%)**			
≤ 60 years	111 (62.7)	208 (60.8)	0.67
> 60 years	66 (37.3)	134 (39.2)	
**Age[a], median (IQR), y**	56 (48-65)	58 (50-65)	0.36
**Sex, No. (%)**			
Male	120 (67.8)	240 (70.2)	0.58
Female	57 (32.2)	102 (29.8)	
**CEA level[b], No. (%)**			
Normal	153 (86.4)	289 (84.5)	0.56
Elevated	24 (13.6)	53 (15.5)	
**CA 19-9 level[c], No. (%)**			
Normal	144 (81.4)	291 (85.1)	0.27
Elevated	33 (18.6)	51 (14.9)	
**Tumor location, No. (%)**			
Fundus of stomach	32 (18.1)	81 (23.7)	0.25
Body of stomach	31 (17.5)	65 (19.0)	
Antrum of stomach	114 (64.4)	196 (57.3)	
**Tumor size, No. (%)**			
≤ 4 cm	86 (45.7)	197 (57.6)	0.06
> 4 cm	91 (54.3)	145 (42.4)	
**Tumor differentiation, No. (%)**			
Well	4 (2.3)	30 (8.8)	<0.001
Moderate	23 (13.0)	84 (24.6)	
Poor and undifferentiated	150 (84.7)	228 (66.7)	
**Lauren type, No. (%)**			
Intestinal type	60 (33.9)	175 (51.5)	<0.001
Diffused or mixed type	117 (66.1)	167 (48.8)	
**Depth of invasion, No. (%)**			
T1	8 (4.5)	83 (24.3)	<0.001
T2	9 (5.1)	37 (10.8)	
T3	12 (6.8)	36 (10.5)	
T4a	129 (72.9)	157 (45.9)	
T4b	19 (10.7)	29 (8.5)	
**Lymph node metastasis, No. (%)**			
N0	50 (28.2)	171 (50.0)	<0.001
N1	37 (20.9)	57 (16.7)	
N2	31 (17.5)	54 (15.8)	
N3a	39 (22.0)	32 (9.4)	
N3b	20 (11.4)	28 (8.1)	

© 2021 Chen D et al. *JAMA Network Open.*

**Distant metastasis, No. (%)**			
M0	159 (89.8)	340 (99.4)	<0.001
M1	18 (10.2)	2 (0.6)	
**TNM stage, No. (%)**			
Stage I	9 (5.1)	92 (26.9)	<0.001
Stage II	43 (24.3)	109 (31.9)	
Stage III	108 (61.0)	138 (40.4)	
Stage IV	17 (9.6)	3 (0.9)	

Abbreviation: $TACS_{GC}$, tumor-associated collagen signature of gastric cancer; IQR, interquartile range; CEA: carcinoembryonic antigen; CA: cancer antigen; TNM, tumor-node-metastasis.

SI conversion factor: To convert CEA to micrograms per liter, multiply by 1.0.

[a] Median difference of age between high- and low-$TACS_{GC}$: 1 (95% confidence interval: -1 to 3) year.

[b] For CEA, elevated indicates 5 ng/mL or greater; normal, less than 5 ng/mL.

[c] For CA 19-9, elevated indicates 37 U/mL or greater; normal, less than 37 U/mL.

© 2021 Chen D et al. *JAMA Network Open.*

eTable 6. Univariate Cox regression analyses of DFS and OS in training cohort

Variables	Disease-free survival		Overall survival	
	**HR (95% CI)**	***P***	**HR (95% CI)**	***P***
**$TACS_{GC}$**[a, b]	3.572 (2.454-5.198)	<0.001	3.542 (2.413-5.199)	<0.001
**Age** (years) (> 60 vs. ≤ 60)	1.258 (0.885-1.788)	0.20	1.301 (0.906-1.868)	0.16
**Sex** (Female vs. Male)	0.706 (0.471-1.057)	0.10	0.746 (0.494-1.128)	0.17
**CEA** (Elevated vs. Normal)	1.809 (1.195-2.737)	0.005	1.914 (1.253-2.922)	0.003
**CA 19-9** (Elevated vs. Normal)	2.195 (1.449-3.325)	<0.001	2.316 (1.515-3.540)	<0.001
**Tumor location**		0.002		0.001
Fundus of stomach	Reference	Reference	Reference	Reference
Body of stomach	0.936 (0.579-1.514)	0.79	0.846 (0.513-1.395)	0.51
Antrum of stomach	0.518 (0.348-0.771)	0.001	0.490 (0.326-0.736)	0.001
**Tumor size** (cm) (> 4 vs. ≤ 4)	2.521 (1.772-3.586)	<0.001	2.680 (1.860-3.863)	<0.001
**Tumor differentiation**		0.03		0.02
Well	Reference	Reference	Reference	Reference
Moderate	5.027 (1.195-21.145)	0.03	4.454 (1.052-18.849)	0.04
Poor and undifferentiated	6.010 (1.482-24.372)	0.01	5.937 (1.463-24.094)	0.01
**Lauren type** (Diffused or mixed vs. Intestinal)	1.621 (1.134-2.317)	0.008	1.727 (1.192-2.503)	0.004
**Depth of invasion**		<0.001		<0.001
T1	Reference	Reference	Reference	Reference
T2	2.083 (0.636-6.825)	0.23	3.034 (0.815-11.300)	0.10
T3	4.743 (1.779-12.644)	0.002	6.469 (2.059-20.330)	0.001
T4a	8.418 (3.680-19.257)	<0.001	11.812 (4.330-32.228)	<0.001
T4b	14.089 (5.496-36.117)	<0.001	21.865 (7.293-65.557)	<0.001
**Lymph node metastasis**		<0.001		<0.001
N0	Reference	Reference	Reference	Reference
N1	2.144 (1.202-3.824)	0.01	2.382 (1.297-4.374)	0.005
N2	4.010 (2.337-6.880)	<0.001	4.554 (2.582-8.032)	<0.001
N3a	9.424 (5.624-15.791)	<0.001	9.540 (5.531-16.454)	<0.001
N3b	11.864 (6.678-21.076)	<0.001	12.963 (7.176-23.417)	<0.001
**Distant metastasis** (M1 vs. M0)	3.671 (1.963-6.866)	<0.001	3.725 (1.990-6.972)	<0.001

Abbreviation: DFS, disease-free survival; OS, overall survival; $TACS_{GC}$, tumor-associated collagen signature of gastric cancer; CEA, carcinoembryonic antigen; CA, cancer antigen; HR, hazard ratio; CI, confidence interval.

[a] Continuous variable.

[b] Per unit increase in 1.

© 2021 Chen D et al. *JAMA Network Open.*

eTable 7. Univariate Cox regression analyses of DFS and OS in validation cohort

Variables	Disease-free survival		Overall survival	
	**HR (95% CI)**	***P***	**HR (95% CI)**	***P***
**$TACS_{GC}$[a, b]**	3.103 (2.256-4.268)	<0.001	3.236 (2.326-4.502)	<0.001
**Age** (years) (> 60 vs. ≤ 60)	1.168 (0.827-1.650)	0.38	1.169 (0.823-1.659)	0.38
**Sex** (Female vs. Male)	1.039 (0.722-1.497)	0.84	1.098 (0.759-1.589)	0.31
**CEA** (Elevated vs. Normal)	1.453 (0.893-2.364)	0.13	1.289 (0.783-2.123)	0.32
**CA 19-9** (Elevated vs. Normal)	2.768 (1.880-4.078)	<0.001	2.992 (2.027-4.418)	<0.001
**Tumor location**		0.18		0.17
Fundus of stomach	Reference	Reference	Reference	Reference
Body of stomach	0.690 (0.404-1.178)	0.17	0.702 (0.409-1.204)	0.20
Antrum of stomach	0.672 (0.438-1.030)	0.07	0.658 (0.426-1.017)	0.06
**Tumor size** (cm) (> 4 vs. ≤ 4)	1.669 (1.179-2.361)	0.004	1.674 (1.176-2.382)	0.004
**Tumor differentiation**		0.008		0.006
Well	Reference	Reference	Reference	Reference
Moderate	1.184 (0.440-3.190)	0.74	1.105(0.408-2.996)	0.84
Poor and undifferentiated	2.328 (0.949-5.710)	0.07	2.284 (0.931-5.604)	0.07
**Lauren type** (Diffused or mixed vs. Intestinal)	1.581 (1.105-2.260)	0.01	1.576 (1.097-2.265)	0.01
**Depth of invasion**		<0.001		<0.001
T1	Reference	Reference	Reference	Reference
T2	3.352 (1.123-10.004)	0.03	3.403 (1.140-10.158)	0.03
T3	4.731 (1.615-13.852)	0.005	4.739 (1.169-13.875)	0.005
T4a	6.028 (2.444-14.868)	<0.001	5.675 (2.298-14.010)	<0.001
T4b	9.263 (3.469-24.735)	<0.001	9.007 (3.374-24.048)	<0.001
**Lymph node metastasis**		<0.001		<0.001
N0	Reference	Reference	Reference	Reference
N1	3.436 (1.931-6.117)	<0.001	3.256 (1.820-5.825)	<0.001
N2	5.815 (3.357-10.073)	<0.001	5.307 (3.049-9.238)	<0.001
N3a	7.108 (4.002-12.622)	<0.001	6.156 (3.454-10.970)	<0.001
N3b	10.467 (5.706-19.202)	<0.001	11.103 (6.050-20.387)	<0.001
**Distant metastasis** (M1 vs. M0)	8.109 (4.028-16.328)	<0.001	8.521 (4.178-17.379)	<0.001

Abbreviation: DFS, disease-free survival; OS, overall survival; $TACS_{GC}$, tumor-associated collagen signature of gastric cancer; CEA, carcinoembryonic antigen; CA, cancer antigen; HR, hazard ratio; CI, confidence interval.

[a] Continuous variable.

[b] Per unit increase in 1.

© 2021 Chen D et al. *JAMA Network Open.*

**eTable 8.** Multivariable Cox regression analyses of DFS and OS without $TACS_{GC}$				
**Variables**	**Disease-free survival**		**Overall survival**	
	**HR (95% CI)**	***P***	**HR (95% CI)**	***P***
**Training cohort**				
**CA 19-9** (Elevated vs. Normal)	1.604 (1.040-2.474)	0.03	1.667 (1.068-2.601)	0.02
**Depth of invasion**		0.004		0.001
T1	Reference	Reference	Reference	Reference
T2	1.478 (0.442-4.945)	0.53	2.115 (0.555-8.053)	0.27
T3	2.740 (1.001-7.499)	0.05	3.261 (1.003-10.602)	0.05
T4a	3.930 (1.635-9.450)	0.002	5.210 (1.823-14.892)	0.002
T4b	5.312 (1.953-14.453)	0.001	8.209 (2.591-26.010)	<0.001
**Lymph node metastasis**		<0.001		<0.001
N0	Reference	Reference	Reference	Reference
N1	1.390 (0.763-2.530)	0.28	1.488 (0.796-2.781)	0.21
N2	2.630 (1.495-4.627)	0.001	2.809 (1.557-5.068)	0.001
N3a	5.252 (3.020-9.133)	<0.001	4.984 (2.801-8.868)	<0.001
N3b	7.541 (4.130-13.769)	<0.001	8.176 (4.398-15.202)	<0.001
**Distant metastasis** (M1 vs. M0)	2.779 (1.431-5.397)	0.003	3.007 (1.543-5.859)	0.001
**Validation cohort**				
**CA 19-9** (Elevated vs. Normal)	1.469 (0.958-2.254)	0.08	1.728 (1.134-2.635)	0.01
**Depth of invasion**		0.02		0.04
T1	Reference	Reference	Reference	Reference
T2	1.913 (0.628-5.829)	0.25	1.912 (0.626-5.838)	0.26
T3	2.644 (0.875-7.991)	0.09	2.479 (0.817-7.521)	0.11
T4a	3.628 (1.441-9.134)	0.006	3.279 (1.301-8.266)	0.01
T4b	4.299 (1.574-11.743)	0.004	3.917 (1.426-10.761)	0.008
**Lymph node metastasis**		<0.001		<0.001
N0	Reference	Reference	Reference	Reference
N1	2.921 (1.624-5.253)	<0.001	2.778 (1.534-5.030)	0.001
N2	4.579 (2.587-8.105)	<0.001	3.966 (2.224-7.074)	<0.001
N3a	4.886 (2.613-9.135)	<0.001	4.043 (2.175-7.516)	<0.001
N3b	7.897 (4.234-14.732)	<0.001	8.029 (4.284-15.048)	<0.001
**Distant metastasis** (M1 vs. M0)	3.189 (1.457-6.977)	0.004	3.388 (1.562-7.351)	0.002

Abbreviation: DFS, disease-free survival; OS, overall survival; $TACS_{GC}$, tumor-associated collagen signature of gastric cancer; CA, cancer antigen; HR, hazard ratio; CI, confidence interval.

© 2021 Chen D et al. *JAMA Network Open.*

eTable 9. C-index comparison of integrated nomogram with other prediction models

**Models**	**Disease-free survival**		**Overall survival**	
	**C-index (95% CI)**	***P***	**C-index (95% CI)**	***P***
**Training cohort**				
Integrated nomogram	0.801 (0.734-0.877)	Reference	0.813 (0.748-0.878)	Reference
Clinicopathological model	0.783 (0.712-0.854)	0.03	0.797 (0.730-0.864)	0.03
TNM stage	0.779 (0.708-0.849)	0.02	0.792 (0.723-0.861)	0.02
$TACS_{GC}$	0.674 (0.580-0.768)	<0.001	0.679 (0.583-0.775)	<0.001
**Validation cohort**				
Integrated nomogram	0.783 (0.699-0.867)	Reference	0.778 (0.694-0.862)	Reference
Clinicopathological model	0.756 (0.668-0.844)	0.006	0.753 (0.671-0.835)	0.002
TNM stage	0.751 (0.665-0.837)	0.001	0.746 (0.664-0.828)	<0.001
$TACS_{GC}$	0.673 (0.581-0.766)	<0.001	0.671 (0.579-0.763)	<0.001

Abbreviation: TNM, tumor-node-metastasis; $TACS_{GC}$, tumor-associated collagen signature of gastric cancer; CI, confidence interval.

© 2021 Chen D et al. *JAMA Network Open.*

eTable 10. The net reclassification improvement of adding the $TACS_{GC}$ to the clinicopathological model

**Cohorts**	**Disease-free survival**		**Overall survival**	
	**NRI (95% CI)**	***P***	**NRI (95% CI)**	***P***
**Integrated nomogram vs. clinicopathological model**				
Training cohort	0.206 (0.047-0.334)	0.01	0.208 (0.019-0.324)	0.03
Validation cohort	0.264 (0.091-0.404)	0.007	0.326 (0.119-0.442)	<0.001

Abbreviation: $TACS_{GC}$, tumor-associated collagen signature of gastric cancer; NRI, net reclassification improvement; CI, confidence interval.

© 2021 Chen D et al. *JAMA Network Open.*

eTable 11. Clinicopathological characteristics of stage II and III patients according to chemotherapy

Variables	No chemotherapy (n=116)	Chemotherapy (n=282)	*P*
**Age, No. (%)**			
≤ 60 years	55 (47.4)	186 (65.9)	0.001
> 60 years	61 (52.6)	96 (34.1)	
**Sex, No. (%)**			
Male	77 (66.4)	200 (70.9)	0.44
Female	39 (33.6)	82 (29.1)	
**CEA level, No. (%)**			
Normal	93 (80.2)	236 (83.7)	0.49
Elevated	23 (19.8)	46 (16.3)	
**CA 19-9 level, No. (%)**			
Normal	89 (76.7)	234 (82.9)	0.19
Elevated	27 (23.3)	48 (17.1)	
**Tumor location, No. (%)**			
Fundus of stomach	31 (26.7)	64 (22.7)	0.65
Body of stomach	24 (20.7)	57 (20.2)	
Antrum of stomach	61 (52.6)	161 (57.1)	
**Tumor size, No. (%)**			
≤ 4 cm	49 (42.2)	140 (49.6)	0.22
> 4 cm	67 (57.8)	142 (50.4)	
**Tumor differentiation, No. (%)**			
Well	4 (3.4)	7 (2.5)	0.45
Moderate	27 (23.3)	52 (18.4)	
Poor and undifferentiated	85 (73.3)	233 (79.1)	
**Lauren type, No. (%)**			
Intestinal type	51 (44.0)	116 (41.1)	0.68
Diffused or mixed type	65 (56.0)	166 (58.9)	
**Depth of invasion, No. (%)**			
T1	3 (2.6)	6 (2.1)	0.77
T2	10 (8.6)	17 (6.0)	
T3	16 (13.8)	31 (11.0)	
T4a	75 (64.7)	196 (69.5)	
T4b	12 (10.3)	32 (11.4)	
**Lymph node metastasis, No. (%)**			
N0	33 (28.4)	89 (31.6)	0.07
N1	26 (22.4)	59 (20.9)	
N2	33 (28.5)	50 (17.7)	
N3a	17 (14.7)	47 (16.7)	
N3b	7 (6.0)	37 (13.1)	
**TNM stage, No. (%)**			
Stage II	45 (38.8)	107 (37.9)	0.96
Stage III	71 (61.2)	175 (62.1)	

Abbreviation: CEA, carcinoembryonic antigen; CA, cancer antigen; TNM, tumor-node-metastasis.

© 2021 Chen D et al. *JAMA Network Open.*

eTable 12. Adjuvant chemotherapy interaction with the $TACS_{GC}$ for DFS and OS in stage II and III patients

$TACS_{GC}$ level	Adjuvant chemotherapy		Disease-free survival			Overall survival		
	**No chemo**	**Chemo**	**HR (95% CI)**	***P***	***P*$_{interaction}$**	**HR (95% CI)**	***P***	***P*$_{interaction}$**
**Stage II and III (n=398)**								
High $TACS_{GC}$ (Chemo vs. No chemo)	47	103	0.796 (0.541-1.170)	0.25	<0.001	0.824 (0.561-1.120)	0.32	<0.001
Low $TACS_{GC}$ (Chemo vs. No chemo)	69	179	0.646 (0.432-0.965)	0.032		0.546 (0.362-0.822)	0.004	
**Stage II (n=152)**								
High $TACS_{GC}$ (Chemo vs. No chemo)	13	30	0.621 (0.259-1.488)	0.29	0.003	0.561 (0.228-1.379)	0.21	0.02
Low $TACS_{GC}$ (Chemo vs. No chemo)	32	77	0.335 (0.121-0.923)	0.034		0.331 (0.119-0.913)	0.033	
**Stage III (n=246)**								
High $TACS_{GC}$ (Chemo vs. No chemo)	34	73	0.928 (0.605-1.424)	0.73	0.004	0.893 (0.582-1.371)	0.61	0.001
Low $TACS_{GC}$ (Chemo vs. No chemo)	37	102	0.610 (0.391-0.952)	0.029		0.479 (0.304-0.756)	0.002	

Abbreviation: DFS, disease-free survival; OS, overall survival; $TACS_{GC}$, tumor-associated collagen signature of gastric cancer; Chemo, chemotherapy; HR, hazard ratio; CI, confidence interval.

© 2021 Chen D et al. *JAMA Network Open.*

eReferences:

1. Dempster A, Laird N, Rubin D. Maximum likelihood from incomplete data via the EM algorithm. *J R Stat Soc B*. 1977; 39(1): 1-38.

2. Stein AM, Vader DA, Jawerth LM, Weitz DA, Sander LM. An algorithm for extracting the network geometry of three-dimensional collagen gels. *J Microsc*. 2008; 232(3): 463-475.

3. Frisch KE, Duenwald-Kuehl SE, Kobayashi H, et al. Quantification of collagen organization using fractal dimensions and Fourier transforms. *Acta Histochem*. 2012; 114(2): 140-144.

4. Daugman JG. Complete discrete 2-D Gabor transforms by neural networks for image analysis and compression. *IEEE Trans Acoust Speech Signal Process*. 1988; 36(7): 1169-1179.

© 2021 Chen D et al. *JAMA Network Open.*
